# Respiratory Syncytial Virus Vaccine Effectiveness Among US Older Adults, 2023-2024 Season

**DOI:** 10.1001/jamanetworkopen.2026.34788

**Published:** 2026-09-18

**Authors:** Yun Lu, Elizabeth R. Smith, Rowan McEvoy, Merianne Rose T. Spencer, Arnstein F. Lindaas, Hector S. Izurieta, Yike Zhang, Henry T. Zhang, Emma Wagner, Mikhail Menis, Nimish Manoj Naik, Whitney R. Steele, Michael Wernecke, Yoganand Chillarige, Steven A. Anderson, Richard A. Forshee

**Affiliations:** 1 Center for Biologics Evaluation and Research, Food and Drug Administration, Silver Spring, Maryland; 2 Acumen LLC, Burlingame, California

## Abstract

**Importance:**

Respiratory syncytial virus (RSV) causes a substantial number of hospitalizations and deaths among older adults. Two RSV vaccines for individuals aged 60 years or older were approved in 2023; it was important to monitor their clinical effectiveness during routine care in the first RSV season after regulatory approval.

**Objective:**

To estimate the clinical effectiveness of RSV vaccines during routine care overall and by brand among US older adults.

**Design, Setting, and Participants:**

This nationally representative, retrospective cohort study evaluated the effectiveness of RSV vaccines from August 6, 2023, to March 2, 2024. The primary analysis included eligible Medicare fee-for-service beneficiaries aged 65 years or older.

**Exposures:**

The RSVPreF3+AS01 RSV vaccine (GSK) and RSVpreF RSV vaccine (Pfizer).

**Main Outcomes and Measures:**

RSV-related hospitalization and RSV-related death.

**Results:**

Among 14 819 226 unvaccinated beneficiaries at the beginning of the study, the median age was 74 years (IQR, 70-79 years) and 57.7% were female. By the study end date, 13.9% of beneficiaries had received a RSVPreF3+AS01 vaccine, and 6.4% received a RSVpreF vaccine. Compared with the unvaccinated cohort, vaccinated individuals were less likely to have low-income status, lived in more affluent areas, and were more likely to have prior influenza vaccination and prior COVID-19 vaccination. The RSVPreF3+AS01 and RSVpreF vaccinated cohorts were largely comparable in demographic, social, and medical characteristics. Overall estimated vaccine effectiveness compared with the unvaccinated cohort was 81.8% (95% CI, 80.0%-83.4%) against RSV-related hospitalization and 85.0% (95% CI, 75.1%-90.9%) against RSV-related death. Brand-specific vaccine effectiveness against RSV-related hospitalization was 79.9% (95% CI, 77.6%-81.9%) for RSVPreF3+AS01 and 85.1% (95% CI, 82.2%-87.5%) for RSVpreF compared with the unvaccinated cohort. Vaccine effectiveness against RSV-related hospitalization was 72.2% (95% CI, 67.4%-76.3%) for immunocompromised individuals. Effectiveness did not change significantly with age.

**Conclusions and Relevance:**

In this cohort study of US older adults, both RSVPreF3+AS01 and RSVpreF vaccines had high effectiveness against RSV-related hospitalization and death during the 2023-2024 season. RSV vaccines demonstrated substantial effectiveness within this high-risk older adult population, including among immunocompromised individuals and across all ages 65 years or older. This large postmarketing noninterventional study provides clinical evidence of RSV vaccine effectiveness in the first year of vaccine approval.

## Key Points

**Question:**

What is the estimated effectiveness of the respiratory syncytial virus (RSV) vaccines against RSV-related hospitalization and death among US adults 65 years or older?

**Findings:**

In this cohort study of 14 819 226 Medicare fee-for-service beneficiaries, both the RSVPreF3+AS01 and RSVpreF RSV vaccines provided substantial protection against RSV-related hospitalization (81.8%) and death (85.0%) during the 2023-2024 season. Effectiveness did not change significantly with age and was also substantial among immunocompromised individuals.

**Meaning:**

This study suggests that both RSV vaccines provided substantial protection among US older adults across all ages 65 years or older and for immunocompromised individuals.

## Teaser

This cohort study estimates the effectiveness of respiratory syncytial virus vaccines in routine care against RSV-related hospitalization and death overall and by brand among US adults aged 65 years or older.

## Introduction

Respiratory syncytial virus (RSV) is a leading cause of lower respiratory tract disease morbidity and mortality, particularly among adults aged 65 years or older and those with compromised immune systems.^1,2^ According to the Centers for Disease Control and Prevention (CDC), RSV contributes to an estimated 60 000 to 160 000 hospitalizations and 6000 to 10 000 deaths annually among US older adults.^2^

In May 2023, the US Food and Drug Administration approved 2 RSV vaccines for individuals 60 years or older: RSVPreF3+AS01 vaccine (Arexvy; GSK) on May 3, 2023,^3^ and RSVpreF vaccine (Abrysvo; Pfizer) on May 31, 2023.^4^ Prelicensure trials had demonstrated high efficacy for these RSV vaccines. An ongoing phase 3 randomized clinical trial (RCT) found RSVPreF3+AS01 significantly reduced the risk of RSV-associated lower respiratory tract disease for adults aged 60 years or older by 82.6% (95% CI, 57.9%-94.1%).^5^ Analyses of an ongoing phase 3 RCT of RSVpreF showed an efficacy of 66.7% (95% CI, 28.8%-85.8%) against first episode of RSV-associated lower respiratory tract disease with 2 or more symptoms and 85.7% (95% CI, 32.0%-98.7%) against first episode of RSV-associated lower respiratory tract disease with 3 or more symptoms among adults aged 60 years or older.^6^

Effectiveness studies of vaccines in routine clinical settings after approval complement the prelicensure RCTs by including high-risk populations, such as very old individuals and immunocompromised individuals, in greater numbers.^7,8^ Three test-negative case-control studies and 1 target trial emulation study found high RSV vaccine effectiveness (VE) for individuals aged 60 years or older.^9,10,11,12^ Additional clinical studies during routine care are needed to assess RSV VE against severe outcomes such as hospitalization and death among older adults, including by age group and immunocompromised status.^7^

Our objective was to estimate the clinical effectiveness of RSV vaccines during routine care against RSV-related hospitalization and death among older adults in the first RSV season after regulatory approval. Moderna’s RSV vaccine was not included in this study as it was approved in May 2024, after the study end date.^13^

## Methods

### Data Sources

Medicare is a federally funded health insurance program covering US individuals aged 65 years or older and those younger than 65 years with certain disabilities or end-stage kidney disease. We used Medicare administrative files, which provide comprehensive data on enrollment, inpatient claims (Part A), hospital outpatient and physician office visit claims (Part B), and prescription drug claims (Part D). Beneficiaries’ demographic and enrollment information was obtained from the Common Medicare Environment. Information on nursing home residency status was ascertained using Medicare Skilled Nursing Facility claims and the Minimum Data Set. The American Community Survey was used to provide information on beneficiaries’ local population density, and Area Deprivation Index (ADI) data from 2019 were used to assess ADI at the block group or zip code level based on the last known address of the beneficiary.^14^

This study was classified as public health surveillance and was exempted from institutional review board approval and informed consent under the common rule at 45 CFR 46.102(l)(2). The use of Medicare administrative data was approved by the Centers for Medicare & Medicaid Services privacy board under a data use agreement. The study followed the Strengthening the Reporting of Observational Studies in Epidemiology (STROBE) reporting guideline for cohort studies. The study protocol is available in Supplement 1.

### Study Population, Study Period, and Study Design

We conducted a retrospective cohort study to assess the VE of RSV vaccines during routine care. The study period was from August 6, 2023, to March 2, 2024. Beneficiary follow-up time began on the first date of estimated high RSV circulation, defined as at least 8 cases per 100 000 beneficiaries in a beneficiary’s census tract on or after October 1, 2023, or from the end of a prior RSV episode, whichever occurred later (eMethods 1 in Supplement 2).

The study population included Medicare fee-for-service (FFS) beneficiaries aged 65 years or older without RSV vaccination prior to the study’s start date. Beneficiaries who lived in a long-term care facility, who were in hospice, or who received care in a dialysis facility were excluded. Additional inclusion and exclusion criteria are listed in eMethods 1 in Supplement 2. We required continuous FFS and pharmacy enrollment from 365 days before the study’s start date through the follow-up start date.

### Exposures and Person Time

The exposures of interest were the receipt of 1 dose of either RSV vaccine (RSVPreF3+AS01 or RSVpreF). Exposures were identified through National Drug Code and Healthcare Common Procedure Coding System codes (eTable 2 in Supplement 2).

Beneficiaries began contributing person-time to the vaccinated cohort 14 days after vaccination and were allowed to switch from the unvaccinated to the vaccinated cohort. We followed beneficiaries until occurrence of the following events: administration of an additional RSV vaccination, disenrollment from Medicare Parts A and B and/or Part D coverage or enrollment into Part C, occurrence of an outcome of interest, death, end of high RSV circulation period, or end of the study period. We listed additional censoring criteria and follow-up requirements in eMethods 1 in Supplement 2.

### Outcomes

The primary outcome was RSV-related hospitalization, identified from inpatient claims using *International Statistical Classification of Diseases and Related Health Problems, Tenth Revision, Clinical Modification*, diagnosis codes B97.4 (respiratory syncytial virus as the cause of diseases classified elsewhere), J12.1 (respiratory syncytial virus pneumonia), J20.5 (acute bronchitis due to respiratory syncytial virus), and J21.0 (acute bronchiolitis due to respiratory syncytial virus). The secondary outcome was RSV-related death: inpatient RSV cases in which the patient died either during the inpatient stay or within 7 or 14 days after inpatient discharge.

We allowed only 1 distinct RSV episode per RSV season. A distinct RSV episode must not have a prior RSV episode that started within 90 days before the start of the current episode or a prior RSV episode that ended within 30 days of the current RSV episode start date.

### Covariates

We adjusted for potential confounders, including beneficiary-level characteristics such as age, sex, race and ethnicity (American Indian or Alaska Native, Asian, Black, Hispanic, White, and other [race and ethnicity not specified] or unknown race), Medicare and Medicaid dual eligibility, general health status (including frailty, immunocompromised status, and presence of specific chronic conditions), prior influenza vaccination status, and prior COVID-19 vaccination status. Race and ethnicity data were not specifically collected for this study; they were ascertained via the Centers for Medicare & Medicaid Services enrollment database and were included as potential confounders. Race and ethnicity is self-reported as a single variable in the Medicare database with the options listed. The study also adjusted for geographic-level variables, including ADI, census tract–level population density, and time-varying census tract–level RSV circulation rate. Analyses evaluated covariate balance across cohorts using standardized mean differences for each week during follow-up. We describe covariates and the algorithm used to identify immunocompromised beneficiaries in eMethods 1 and eTable 1 in Supplement 2.

### Statistical Analysis

We conducted cohort comparisons overall and by vaccine brand. For each vaccine cohort, we summarized the outcome counts, person-time distribution, and unadjusted outcome rates. We used Poisson regressions to estimate the rate ratios (RRs) for RSV-related outcomes in the vaccinated cohort compared with the unvaccinated cohort, and used marginal structural models to address time-varying exposures.^15,16^ Specifically, we estimated the average treatment effect using inverse probability weighting to adjust for factors potentially associated with vaccination status and RSV risk among individuals, where the overall weight was calculated using 2 time-varying weights estimating probability of treatment and censoring across follow-up time. We then ran a Poisson model with weekly interval–specific intercepts on the propensity score–weighted pseudopopulation to estimate RRs for each of the cohort comparisons.^17^ This discrete time model provides a close approximation to a Cox proportional hazards regression model because follow-up was partitioned into fine intervals, and the interval intercepts captured the baseline hazard. VE was calculated as VE = [100 × (1 − RR)]%.

To estimate VE, we ran an outcome model including brand-specific exposures (eMethods 2 in Supplement 2). To reduce the impact of residual confounding on VE estimates, we reported doubly robust results with propensity score model covariates included in the outcome model. Statistical significance was determined by 2-sided hypothesis tests at the *P* < .05 level.

We performed subgroup analyses by immunocompromised status, with post hoc analyses by specific immunocompromising subcategories. We conducted sensitivity analyses with alternate RSV circulation thresholds (ie, no threshold, and a threshold of 16 cases per 100 000 beneficiaries) as well as removing the requirement of on or after October 1, 2023, for inclusion of follow-up time in the model. To investigate the potential association of health-seeking behavior with VE estimates, we performed analyses restricted to individuals with prior influenza vaccination (a surrogate for health-seeking behavior) within 365 days prior to study start date. We additionally used splines to model potential nonlinear associations of RSV circulation in the propensity score model, and of RSV circulation and age in the outcome model (eTable 5 in Supplement 2). We also evaluated the association of age with VE estimates by including an age-RSV vaccination interaction term. For all analyses using splines, we compared linear models as well as models with 1– or 2–interior-knot natural splines and selected the model with the lowest Akaike Information Criterion.^18^ For all analyses, we used R, version 4.4.0 (R Project for Statistical Computing), and SAS, version 9.4 (SAS Institute Inc).

## Results

### Descriptive Analyses

**Figure 1.**
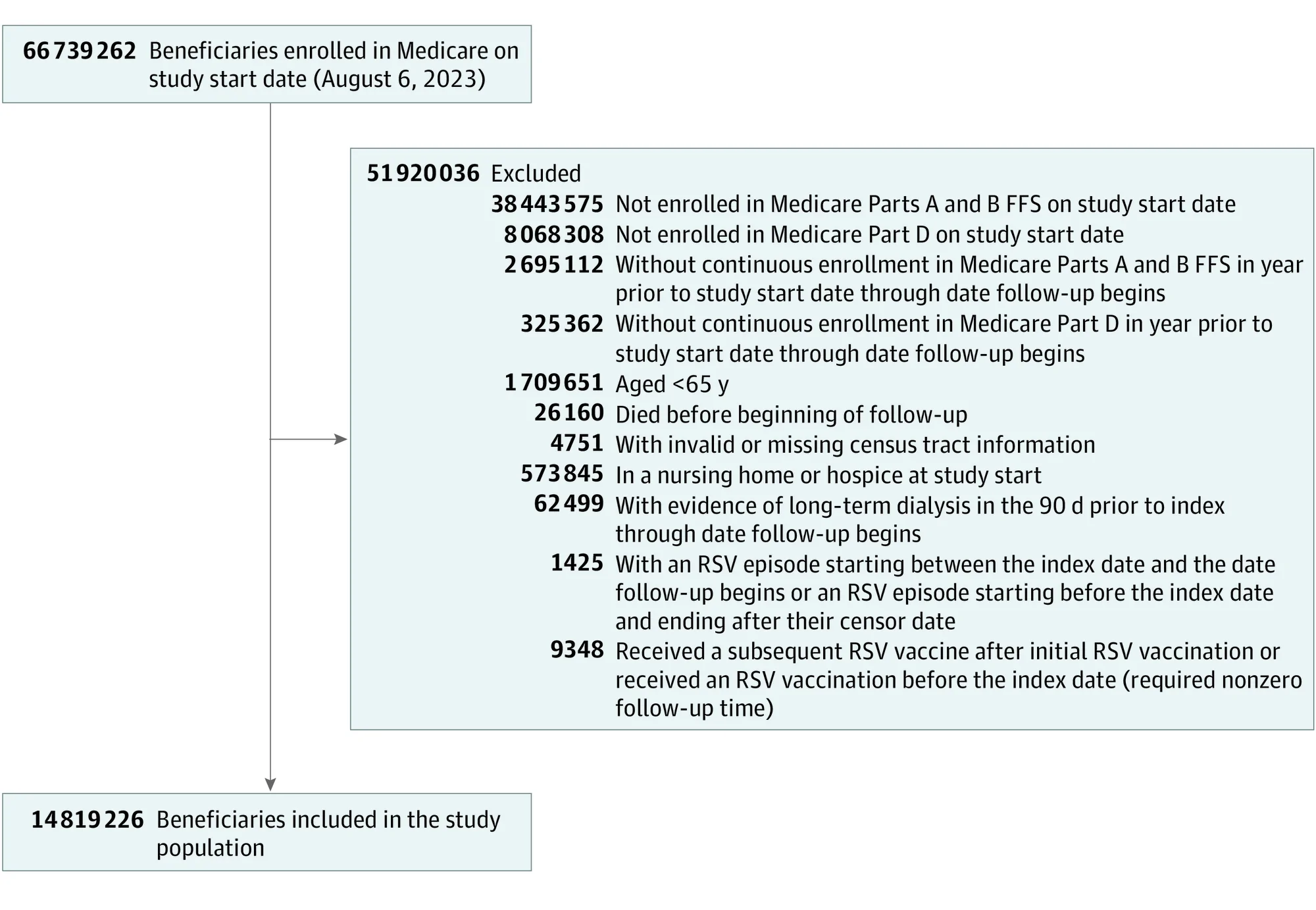
Flow Diagram of Study Population Formation FFS indicates Fee-For-Service; and RSV, respiratory syncytial virus.

The study included 14 819 226 eligible community-dwelling Medicare FFS beneficiaries aged 65 years or older (median age, 74 years [IQR, 70-79 years]; 57.7% female and 42.3% male; 0.3% American Indian or Alaska Native, 2.3% Asian, 4.5% Black, 1.5% Hispanic, 86.7% White, and 4.8% other or unknown race) (Figure 1 and Table 1). Among them, 2 064 913 (13.9%) had received a RSVPreF3+AS01 vaccine and 955 607 (6.4%) had received a RSVpreF vaccine before March 2, 2024 (Table 1). Compared with the unvaccinated cohort, vaccinated individuals were less likely to have low-income status (Medicare and Medicaid dual eligibility, 4.2% vs 12.9%), lived in more affluent areas (median ADI, 33 [IQR, 16-55] vs 41 [IQR, 21-64]), and were more likely to have prior influenza vaccination (90.6% vs 56.4%) and prior COVID-19 vaccination (77.9% vs 33.9%). Population characteristics were similar between vaccine brands. After weighting, cohorts were largely balanced on covariates included in weighting except census tract–level RSV circulation rates. Prior influenza vaccination status and prior COVID-19 vaccination status (not included in the propensity score model given their close association with the exposures, but included in the outcome model) were also unbalanced.

**Table 1.** Covariate Distribution Across RSV Vaccine Cohorts

Covariate^b^	Cohort, No. (%)			Maximum SMD^a^	
	RSV unvaccinated	RSV vaccinated: RSVpreF	RSV vaccinated: RSVPreF3+AS01	Unweighted SMD	Weighted SMD
Population, No.	11 798 706	955 607	2 064 913	NA	NA
Sex					
Female	6 821 894 (57.8)	549 246 (57.5)	1 185 642 (57.4)	0.01	0.01
Male	4 976 812 (42.2)	406 361 (42.5)	879 271 (42.6)	0.01	0.01
Age, y					
65-69	2 902 712 (24.6)	180 594 (18.9)	397 171 (19.2)	0.14	0.00
70-74	3 446 909 (29.2)	291 532 (30.5)	635 528 (30.8)	0.03	0.01
75-79	2 500 102 (21.2)	239 875 (25.1)	521 634 (25.3)	0.10	0.00
80-84	1 590 708 (13.5)	145 227 (15.2)	308 489 (14.9)	0.05	0.01
≥85	1 358 275 (11.5)	98 379 (10.3)	202 091 (9.8)	0.06	0.02
CMS Medicare race and ethnicity					
American Indian or Alaska Native	38 811 (0.3)	1950 (0.2)	2299 (0.1)	0.05	0.02
Asian	281 326 (2.4)	13 748 (1.4)	40 185 (1.9)	0.07	0.01
Black	588 677 (5.0)	23 695 (2.5)	54 214 (2.6)	0.13	0.03
Hispanic	203 230 (1.7)	3500 (0.4)	8900 (0.4)	0.13	0.01
White	10 147 746 (86.0)	863 710 (90.4)	1 839 659 (89.1)	0.14	0.02
Other or unknown race^c^	538 915 (4.6)	49 004 (5.1)	119 655 (5.8)	0.06	0.01
Medicare and Medicaid dual eligibility					
Dual	1 516 787 (12.9)	39 078 (4.1)	86 377 (4.2)	0.32	0.02
Nondual	10 281 919 (87.1)	916 529 (95.9)	1 978 536 (95.8)	0.32	0.02
Reason for entering Medicare					
Aged in without ESKD	10 721 937 (90.9)	895 471 (93.7)	1 940 400 (94.0)	0.12	0.04
Disabled without ESKD	1 064 894 (9.0)	59 106 (6.2)	122 349 (5.9)	0.12	0.04
ESKD only	11 875 (0.1)	1030 (0.1)	2164 (0.1)	0.00	0.00
Frailty conditions					
Depression	2 069 847 (17.5)	194 066 (20.3)	405 807 (19.7)	0.07	0.02
Parkinson disease	170 729 (1.4)	14 036 (1.5)	29 366 (1.4)	0.00	0.01
Arthritis	3 941 171 (33.4)	368 024 (38.5)	789 467 (38.2)	0.11	0.02
Cognitive impairment	1 369 101 (11.6)	98 437 (10.3)	208 168 (10.1)	0.05	0.02
Paranoia	1 733 786 (14.7)	157 888 (16.5)	327 496 (15.9)	0.05	0.02
Chronic skin ulcer	371 821 (3.2)	27 740 (2.9)	58 584 (2.8)	0.02	0.01
Skin and soft tissue infection	926 455 (7.9)	82 568 (8.6)	180 512 (8.7)	0.03	0.02
Mycoses	1 675 719 (14.2)	153 015 (16.0)	327 311 (15.9)	0.05	0.04
Gout	654 169 (5.5)	56 403 (5.9)	124 057 (6.0)	0.02	0.01
Falls	703 131 (6.0)	58 319 (6.1)	122 442 (5.9)	0.01	0.01
Musculoskeletal problems	6 760 221 (57.3)	613 614 (64.2)	1 322 841 (64.1)	0.14	0.03
UTI	1 692 342 (14.3)	132 150 (13.8)	294 234 (14.2)	0.01	0.03
Pneumonia	490 034 (4.2)	46 396 (4.9)	99 124 (4.8)	0.03	0.01
Charlson Comorbidity Index score >0	7 371 559 (62.5)	632 454 (66.2)	1 367 831 (66.2)	0.08	0.05
Hospital admission in past 6 mo	823 577 (7.0)	64 763 (6.8)	138 020 (6.7)	0.01	0.02
Prior RSV diagnosis	16 694 (0.1)	2611 (0.3)	5334 (0.3)	0.03	0.00
Prior vaccinations					
Influenza vaccination	6 649 643 (56.4)	864 390 (90.5)	1 871 300 (90.6)	0.84	0.78
COVID-19 vaccination	3 996 223 (33.9)	743 159 (77.8)	1 608 338 (77.9)	0.99	0.90
Immunocompromised status	1 030 766 (8.7)	108 090 (11.3)	234 837 (11.4)	0.09	0.02
Presence of medical conditions					
Chronic kidney disease	2 070 439 (17.5)	173 053 (18.1)	374 406 (18.1)	0.02	0.02
Chronic liver disease	767 779 (6.5)	66 085 (6.9)	148 807 (7.2)	0.03	0.02
Diabetes	3 242 637 (27.5)	247 925 (25.9)	540 038 (26.2)	0.03	0.03
Neurologic or neurodevelopmental conditions	6 270 941 (53.1)	570 133 (59.7)	1 219 671 (59.1)	0.13	0.02
Obesity	2 946 029 (25.0)	253 736 (26.6)	544 781 (26.4)	0.04	0.03
Hypertension					
Essential hypertension without complicated hypertension or other CVD	4 709 259 (39.9)	392 686 (41.1)	849 567 (41.1)	0.03	0.01
Essential hypertension with other CVD or complicated hypertension	3 407 154 (28.9)	286 620 (30.0)	619 191 (30.0)	0.02	0.04
Other CVD					
Atrial fibrillation	1 656 698 (14.0)	148 037 (15.5)	315 510 (15.3)	0.04	0.02
Congestive heart failure	1 324 058 (11.2)	106 717 (11.2)	223 656 (10.8)	0.01	0.02
Coronary revascularization	113 462 (1.0)	8916 (0.9)	19 242 (0.9)	0.00	0.00
Hospitalized AMI	87 510 (0.7)	5895 (0.6)	12 325 (0.6)	0.02	0.00
Hospitalized stroke or TIA	68 352 (0.6)	4193 (0.4)	9091 (0.4)	0.02	0.00
Other cerebrovascular disease	1 013 239 (8.6)	80 757 (8.5)	181 018 (8.8)	0.01	0.02
Other chronic respiratory diseases					
Asthma without COPD	616 069 (5.2)	77 722 (8.1)	170 686 (8.3)	0.12	0.00
Bronchiectasis	121 442 (1.0)	18 541 (1.9)	43 111 (2.1)	0.09	0.01
COPD	1 253 744 (10.6)	120 497 (12.6)	256 034 (12.4)	0.06	0.02
Hypersensitivity pneumonitis	118 574 (1.0)	9755 (1.0)	20 349 (1.0)	0.00	0.01
Interstitial lung disease	109 311 (0.9)	14 564 (1.5)	31 464 (1.5)	0.05	0.01
ADI, national percentile					
1st-10th	1 412 819 (12.0)	137 508 (14.4)	378 519 (18.3)	0.18	0.02
11th-20th	1 439 118 (12.2)	131 141 (13.7)	312 070 (15.1)	0.08	0.02
21st-30th	1 492 703 (12.7)	136 418 (14.3)	306 492 (14.8)	0.06	0.01
31st-40th	1 489 256 (12.6)	131 501 (13.8)	273 586 (13.2)	0.03	0.01
41st-50th	1 352 479 (11.5)	113 901 (11.9)	221 496 (10.7)	0.04	0.01
51st-60th	1 222 845 (10.4)	93 653 (9.8)	178 043 (8.6)	0.06	0.01
61st-70th	1 084 708 (9.2)	76 903 (8.0)	144 665 (7.0)	0.08	0.01
71st-80th	960 213 (8.1)	63 044 (6.6)	115 329 (5.6)	0.10	0.00
81st-90th	814 138 (6.9)	45 876 (4.8)	85 608 (4.1)	0.12	0.01
91st-100th	522 982 (4.4)	25 293 (2.6)	48 132 (2.3)	0.12	0.02
Missing	7445 (0.1)	369 (0.0)	973 (0.0)	0.01	0.00
Census tract–level population density: continuous log base 2, mean (SD)	9.6 (3.0)	10.1 (2.7)	9.8 (2.8)	0.07	0.06
RSV circulation (2-wk rate): continuous log base 2, per 100 000, mean (SD)	4.5 (0.7)	4.4 (0.7)	4.4 (0.7)	0.15	0.07

Abbreviations: ADI, Area Deprivation Index; AMI, acute myocardial infarction; CMS, Centers for Medicare & Medicaid Services; COPD, chronic obstructive pulmonary disease; CVD, cardiovascular disease; ESKD, end-stage kidney disease; NA, not applicable; RSV, respiratory syncytial virus; SMD, standardized mean difference; TIA, transient ischemic attack; UTI, urinary tract infection.

^a^ SMD values of greater than or equal to 0.10 indicate that the groups are imbalanced on the specified covariates. SMDs are pairwise comparisons conducted between cohorts. Only the maximum SMD is displayed here.

^b^ Covariate distribution was evaluated at the end of individual follow-up time.

^c^ Other race and ethnicity not specified.

Prior to weighting, there were 12 569 RSV-related hospitalizations in the unvaccinated population, 352 hospitalizations in the RSVPreF3+AS01 cohort, and 134 hospitalizations in the RSVpreF cohort (Table 2). Prior to weighting, there were 399 inpatient deaths in the unvaccinated population and 12 inpatient deaths among all vaccinated beneficiaries. When including deaths within 7 days after discharge, there were a total of 477 deaths in the unvaccinated population and 16 deaths among all vaccinated beneficiaries; when including deaths within 14 days after discharge, there were a total of 503 deaths in the unvaccinated population and 17 deaths among all vaccinated beneficiaries. Number of patients by censoring reasons and follow-up time distributions are shown in eTable 3 and eTable 4 in Supplement 2.

**Table 2.** Unweighted and Weighted Outcome Rates

Outcome: cohort	Unweighted			Weighted		
	Outcomes, No.	Total person-time, ×100 000 person-wk	Outcome rate (95% CI)	Outcomes, No.	Total person-time, ×100 000 person-wk	Outcome rate (95% CI)
**RSV-related hospitalization**						
Unvaccinated	12 569	2220.73	5.66 (5.56-5.76)	13 051.04	2223.88	5.87 (5.77-5.97)
RSVPreF3+AS01	352	273.14	1.29 (1.15-1.42)	352.94	267.96	1.32 (1.18-1.45)
RSVpreF	134	128.64	1.04 (0.87-1.22)	128.29	124.27	1.03 (0.85-1.21)
**RSV-related death, during inpatient stay**						
Unvaccinated	399	2221.65	0.18 (0.16-0.20)	423.03	2224.82	0.19 (0.17-0.21)
Vaccinated	12	401.84	0.03 (0.01-0.05)	10.84	392.29	0.03 (0.01-0.04)
**RSV-related death, 7 d after inpatient discharge**						
Unvaccinated	477	2221.65	0.21 (0.20-0.23)	502.13	2224.82	0.23 (0.21-0.25)
Vaccinated	16	401.84	0.04 (0.02-0.06)	15.26	392.29	0.04 (0.02-0.06)
**RSV-related death, 14 d after inpatient discharge**						
Unvaccinated	503	2221.65	0.23 (0.21-0.25)	529.98	2224.82	0.24 (0.22-0.26)
Vaccinated	17	401.84	0.04 (0.02-0.06)	16.74	392.29	0.04 (0.02-0.06)

Abbreviation: RSV, respiratory syncytial virus.

### Primary Analyses

**Figure 2.**
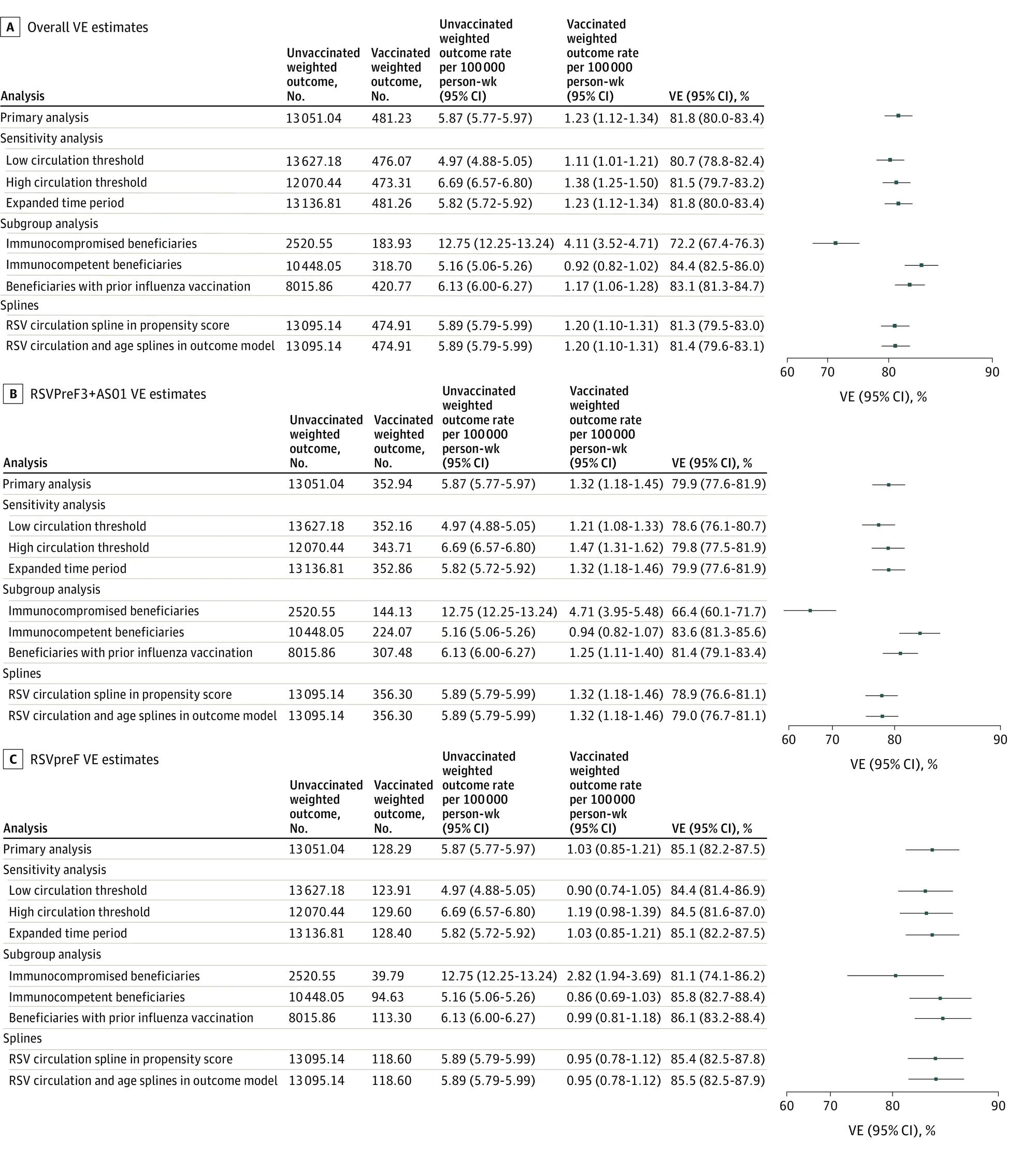
Forest Plots of Vaccine Effectiveness (VE) Estimates and Respiratory Syncytial Virus (RSV)–Related Hospitalization Outcomes A, Overall VE Estimates against RSV-related hospitalization outcomes. B, RSVPreF3+AS01 VE estimates against RSV-related hospitalization outcomes. C, RSVpreF VE estimates against RSV-related hospitalization outcomes.

**Figure 3.**
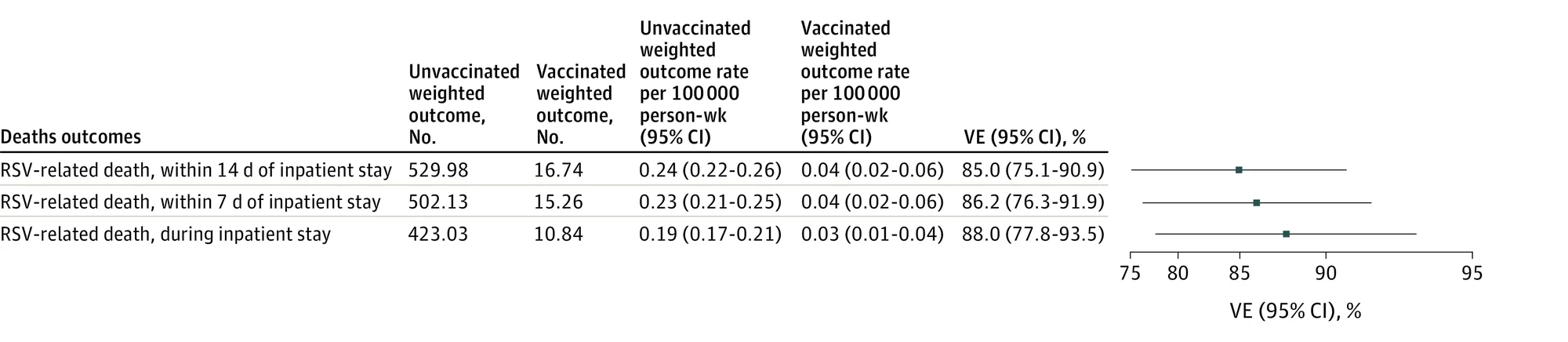
Forest Plot of Vaccine Effectiveness (VE) Estimates and Respiratory Syncytial Virus (RSV)–Related Death Outcomes

Compared with the unvaccinated cohort, overall VE was 81.8% (95% CI, 80.0%-83.4%) against RSV-related hospitalization and 85.0% (95% CI, 75.1%-90.9%) against RSV-related death (Figure 2 and Figure 3). Brand-specific VE against RSV-related hospitalization was 79.9% (95% CI, 77.6%-81.9%) for RSVPreF3+AS01 and 85.1% (95% CI, 82.2%-87.5%) for RSVpreF (Figure 2).

### Alternate Circulation Threshold Sensitivity Analysis

Modifying the high RSV circulation threshold showed similar results as the primary analysis. For the hospitalization outcome, removing the RSV circulation threshold requirement resulted in VEs of 78.6% (95% CI, 76.1%-80.7%) for RSVPreF3+AS01 and 84.4% (95% CI, 81.4%-86.9%) for RSVpreF (Figure 2). Increasing the threshold to 16 cases per 100 000 beneficiaries resulted in VEs of 79.8% (95% CI, 77.5%-81.9%) for RSVPreF3+AS01 and 84.5% (95% CI, 81.6%-87.0%) for RSVpreF.

### Prior Influenza Vaccine Subgroup Analysis

Results among beneficiaries with a prior influenza vaccination were similar to the primary analysis: overall VE against RSV-related hospitalization was 83.1% (95% CI, 81.3%-84.7%) (Figure 2). Brand-specific VE against RSV-related hospitalization was 81.4% (95% CI, 79.1%-83.4%) for RSVPreF3+AS01 and 86.1% (95% CI, 83.2%-88.4%) for RSVpreF.

### Spline Analyses

The model with spline for RSV circulation included in the propensity score showed VE estimates consistent with the primary analysis, as did the analysis including 2-knot splines for age and RSV circulation in the outcome model (Figure 2). Interacting age spline and RSV vaccination showed no evidence of effect modification by age (eTable 6 and the eFigure in Supplement 2).

### Subgroup Analyses by Immunocompromised Status

Overall VE against RSV-related hospitalization was 84.4% (95% CI, 82.5%-86.0%) for immunocompetent beneficiaries and 72.2% (95% CI, 67.4%-76.3%) for immunocompromised individuals (Figure 2). For immunocompetent beneficiaries, brand-specific effectiveness against RSV-related hospitalization was 83.6% (95% CI, 81.3%-85.6%) for RSVPreF3+AS01 and 85.8% (95% CI, 82.7%-88.4%) for RSVpreF. For immunocompromised beneficiaries, brand-specific effectiveness against RSV-related hospitalization was 66.4% (95% CI, 60.1%-71.7%) for RSVPreF3+AS01 and 81.1% (95% CI, 74.1%-86.2%) for RSVpreF. VE results by immunocompromised subcategories are shown in eTable 7 in Supplement 2.

## Discussion

In this nationally representative RSV VE study of nearly 15 million US older adults, both the RSVPreF3+AS01 and RSVpreF RSV vaccines demonstrated high estimated effectiveness against RSV-related hospitalization and death during the first season in which the vaccines were available. Our findings are consistent with results from prelicensure randomized clinical trials and other US noninterventional studies for adults aged 60 years or older, including 3 test-negative case-control studies and a target trial emulation study.^5,6,9,10,11,12^ Our study’s large number of vaccinated older adults (approximately 2 million receiving RSVPreF3+AS01 and 0.9 million receiving RSVpreF) allowed us to generate effectiveness estimates against death. To our knowledge, this is the first study to assess VE against RSV-related death. We found the vaccines highly protective against death during RSV-related hospitalizations as well as within 7 days and 14 days after RSV-related hospitalizations, as there were fewer than 20 RSV-related deaths among 3 million vaccinated individuals. The large study size allowed us to granularly assess risk against RSV-related hospitalization by age, finding no evidence of effect modification by age, including for beneficiaries aged 85 years or older. The large study size also allowed us to assess effectiveness by more granular immunocompromised subcategories. RSV vaccines provided substantial protection to immunocompromised individuals.

Any interpretation of RSV VE in the routine clinical setting is more challenging in this first season due to the shared clinical decision-making recommendation for RSV vaccine receipt.^7^ The CDC’s Advisory Committee on Immunization Practices initially recommended in June 2023 that adults aged 60 years or older should make the decision regarding RSV vaccination with their health care providers (defined by the CDC as anyone who provides or administers vaccines: primary care physicians, specialists, physician assistants, nurse practitioners, registered nurses, and pharmacists).^19^ This shared-decision model (later updated by the CDC in June 2024^19^) could introduce confounding by creating differences between the cohorts. On one hand, health care providers may recommend RSV vaccines to more frail individuals, who are at greater risk of severe RSV outcomes, and to individuals with higher exposure to RSV, resulting in a potential underestimation of the VE. On the other hand, healthier individuals who tend to seek health care may be more likely to choose to be vaccinated, and they may also be more likely to seek care when sick. The lower baseline risk of healthier individuals could result in an overestimation of VE, while the health-seeking behavior difference could result in an underestimation of the VE.

Before conducting the study, we first assessed fitness for use (reliability and relevance) of the Medicare claims data and evaluated potential sources of bias.^20,21^ As RSV vaccinations are administered largely in the pharmacy setting, there is accurate exposure capture in Part D data. However, outcome misclassification could be an issue, given that RSV outcomes have historically been undercaptured. Previous studies have suggested that influenza and COVID-19 diagnosis codes in medical claims may be more reliable during high circulation periods and have lower false-positive rates.^22,23,24^ A similar phenomenon could have happened with RSV. In addition, health-seeking behavior could be a potential confounder because a record in “medical claims databases is generated only if there is an interaction of the patient with the health care system.”^20^

We worked to minimize bias in the study design stage.^21^ To avoid immortal time bias, we used time-varying exposures and allowed a person to switch from the unvaccinated to vaccinated cohort. To reduce the impact of outcome misclassification, this study focused on severe outcomes such as RSV-related hospitalization and death, which are less likely to be misclassified.^25^ In addition, severe outcomes are less impacted by health-seeking behavior. To minimize potential bias due to false-positive RSV outcomes, we limited our primary analysis to census tracts with high RSV circulation. We also planned sensitivity analyses investigating the association of different high RSV circulation parameters with the VE estimates and found our results were robust to selection of different high circulation thresholds, which were different from observations in previous influenza and COVID-19 VE studies.

We also controlled for bias in the analysis stage.^21^ We used propensity score weighting to control for demographic characteristics, socioeconomic status, medical conditions, and time-varying factors such as RSV circulation, and used a doubly robust approach to include all propensity score covariates in the outcome model. To control for health-seeking behavior, we conducted subgroup analyses among beneficiaries who had prior influenza vaccination. Previous studies have used prior influenza vaccination status as a surrogate for a person’s health-seeking behavior.^26^ We observed cohort imbalances regarding prior influenza vaccinations, with a much higher percentage of prior influenza vaccination among individuals who received RSV vaccination (approximately 91%) compared with those who did not (approximately 56%). The subgroup analyses of individuals with prior influenza vaccination generated very similar VE estimates as the primary analyses, suggesting that our results were robust with respect to health-seeking behavior differences. The consistency of our findings using both approaches may be attributed to our use of propensity score weighting to improve cohort balance, use of adjustments for prior vaccinations in the outcome model, and the fact that severe outcomes such as hospitalizations and death should be less impacted by health-seeking behavior differences.

We used the Centers for Medicare & Medicaid Services Medicare FFS database, a large, nationally representative database with minimal beneficiary attrition, ensuring highly generalizable findings to the US older adult population. This enabled evaluation of very rare outcomes, including death. Our study simultaneously adjusted for many time-invariant confounders and time-varying RSV circulation and population density at the census tract level. We had sufficient power to also conduct subgroup analyses among very high-risk populations such as immunocompromised individuals, typically excluded from prelicensure RCTs.

### Limitations

Our study has several limitations. First, we lacked laboratory-confirmed diagnoses of RSV infection, and RSV diagnoses are potentially underreported in claims data. This study should not be interpreted as analyzing RSV disease burden, and we did not report absolute risk reduction because it would likely be an underestimate. However, outcome misclassification would likely not be associated with RSV vaccination status and thus should not affect our VE results in any major way. Second, we investigated hospitalizations and deaths after RSV diagnoses in Medicare claims data but could not confirm that the direct cause of those outcomes was RSV infection. However, prior studies have shown a high positive predictive value for RSV hospital discharge diagnoses.^25^ Third, our study was limited to the Medicare population aged 65 years or older and did not include beneficiaries aged 60 to 64 years. Nonetheless, our VE estimates were very similar to those of other studies in adults aged 60 years or older.

## Conclusions

This nationally representative retrospective cohort study of US adults aged 65 years or older demonstrated that both the RSVPreF3+AS01 and RSVpreF RSV vaccines had high estimated effectiveness against RSV-related hospitalization and death. RSV vaccines demonstrated substantial effectiveness within this high-risk population of older adults, including among immunocompromised individuals and across all ages 65 years or older. This large postmarketing noninterventional study provides clinical evidence of RSV vaccine effectiveness in the first year of vaccine approval.

## Notes

Supplement 1.
Study Protocol

Supplement 2. **eMethods 1.** Population Construction **eTable 1.** List of Covariates **eMethods 2.** Statistical Analyses **eTable 2.** Vaccine Codes for Approved Respiratory Syncytial Virus (RSV) Vaccines **eTable 3.** Censoring Reason Summary **eTable 4.** Distribution of Follow Up Time Among Study Participants, Days **eTable 5.** Specifications for Age Investigation **eTable 6.** VE Estimates for Best Fitting Model With Age-Interaction Term: 2-Interior Knot Age Spline and Linear Age Interaction Term **eFigure.** VE Estimates for Best Fitting Model With Age-Interaction Term: 2-Interior Knot Age Spline and Linear Age Interaction Term **eTable 7.** VE Results by Immunocompromised Subcategories

Supplement 3.
Data Sharing Statement
