# Supplementary material for: Respiratory Syncytial Virus Vaccine Effectiveness Among US Older Adults, 2023-2024 Season
Source: JAMA Netw Open. 2026 Sep 18;9(9):e2634788. doi: 10.1001/jamanetworkopen.2026.34788 (PMC13589284; doi:10.1001/jamanetworkopen.2026.34788)
Supplement: Supplementary file 1 [file jamanetwopen-e2634788-s001.pdf]

# Absolute and Relative Effectiveness of Respiratory Syncytial Virus (RSV) Vaccines Among Community-Dwelling Medicare Beneficiaries Aged ≥65 years

## Protocol

**Protocol Date: 05/22/2024**


**Prepared by**

**FDA:** Yun Lu, Richard A. Forshee, Mikhail Menis, Merianne R. Spencer, Henry T. Zhang, Steven A. Anderson, Whitney R. Steele

**ACUMEN, LLC:** Ellie Smith, Vincent Varvaro, Rowan McEvoy, Easheta Shah, Emma Wagner, Olivia Zhang, Nimish Naik, Mike Wernecke, Arnstein Lindaas, Yoganand Chillarige

## VERSION CONTROL

| Name | Date | Version | Description |
|---|---|---|---|
| Draft 1.0 | March 6, 2024 | 1.0 | Initial Draft |
| Draft 2.0 | April 9, 2024 | 1.1 | Draft 2 |
| Draft 3.0 | April 23, 2024 | 1.2 | Draft 3 |
| Draft 4.0 | May 8, 2024 | 1.3 | Draft 4 |
| Final Draft | May 22, 2024 | 1.4 | Final Draft |

**Table of Contents**


Protocol ........ 1

1. BACKGROUND ........ 3

2. OBJECTIVES ........ 3

2.1 Primary Objectives ........ 3

2.2 Secondary Objectives ........ 3

2.3 Follow-up Study ........ 4

3. METHODS ........ 4

3.1 Study Design ........ 4

3.2 Data Sources ........ 4

3.3 Study Period ........ 5

3.4 Exposure and Index Date Definition ........ 5

3.5 Study Population ........ 6

3.6 Follow-up Time and Censoring ........ 6

3.7 Outcome Definitions ........ 7

3.8 Covariate Summary ........ 7

3.8.1 Time-Invariant Covariates ........ 8

3.8.2 Time-Varying Covariates ........ 8

3.9 Statistical Analysis ........ 10

3.9.1 Marginal Structural Models ........ 10

3.9.2 Descriptive Analyses ........ 10

3.9.3 Covariate Balancing Method ........ 11

3.9.4 Primary Analysis ........ 11

3.9.5 Secondary Analyses ........ 12

3.9.6 Follow Up Analyses ........ 13

4. ACKNOWLEDGEMENT AND CERTIFICATION ........ 14

5. REFERENCES ........ 15

6. APPENDIX ........ 16

A. Immunocompromised Algorithm ........ 16

B. RSV Vaccine Codes ........ 17

C. Marginal Structural Models Statistical Implementation ........ 18

# 1. BACKGROUND

Respiratory syncytial virus (RSV) infection in older adults (individuals aged ≥65 years) can result in Lower Respiratory Tract Disease (LRTD), which can lead to serious, life-threatening pneumonia and bronchiolitis.[1] According to the Centers for Disease Control and Prevention (CDC), RSV infection causes approximately 60,000 – 120,000 hospitalizations and 6,000 – 10,000 deaths annually among adults aged 65 years and older.[2] The United States (U.S.) Food and Drug Administration (FDA) approved two RSV vaccines for the prevention of LRTD caused by RSV infection in individuals 60 years of age and older: The GSK RSVPreF3 (AREXVY) RSV vaccine was approved on May 3, 2023 and the Pfizer RSVpreF (ABRYSVO) RSV vaccine was approved on May 31, 2023.[1,3] Following FDA approval of both vaccines, the CDC endorsed recommendations that adults 60 years of age and older may receive a single dose of an RSV vaccine based on a shared clinical decision-making model, meaning that "health care providers and their patients should have a conversation to determine if RSV vaccination will be beneficial".[4]

This protocol outlines a study designed to assess the real-world effectiveness of both approved RSV vaccines in the United States.

# 2. OBJECTIVES

The objective of this study is to evaluate the effectiveness of Pfizer RSVpreF and GSK RSVPreF3 RSV vaccines for preventing RSV outcomes among community-dwelling Medicare beneficiaries aged ≥65 years.

## 2.1 Primary Objectives

The primary objectives are to evaluate:

i. The absolute effectiveness of Pfizer RSVpreF and GSK RSVPreF3 for preventing RSV-related hospitalization among community-dwelling Medicare beneficiaries aged ≥65 years.

This protocol describes the primary analysis for the primary objective: follow-up analyses for the primary objectives as well as the secondary objectives will be specified in later documents.

## 2.2 Secondary Objectives

The secondary objectives are to evaluate:

i. The absolute effectiveness of Pfizer RSVpreF and GSK RSVPreF3 for preventing RSV-related outcomes.

ii. The relative effectiveness of Pfizer RSVpreF relative to GSK RSVPreF3 for preventing RSV-related outcomes.
iii. The impact of time since vaccination on the absolute effectiveness of vaccination for preventing RSV-related outcomes.
iv. The impact of time since vaccination on the relative effectiveness of Pfizer RSVpreF relative to GSK RSVPreF3 for preventing RSV-related outcomes.

### 2.3 Follow-up Study

The objectives of a follow-up study, pending results from primary and secondary analyses are to evaluate:

i. The absolute and relative effectiveness of Pfizer RSVpreF and GSK RSVPreF3 RSV vaccines for preventing RSV-related outcomes in nursing home beneficiaries.

## 3. METHODS

### 3.1 Study Design

This study will employ a retrospective cohort design using a marginal structural model.[5] The primary analysis will be conducted on an individual level and individuals will be allowed to change cohorts throughout the study period to reflect changing vaccination status.

### 3.2 Data Sources

The primary data sources for this study are Medicare enrollment records and claims data from Centers for Medicare and Medicaid Services (CMS). Medicare is a national health insurance program that provides healthcare coverage for people aged ≥65 years, as well as those with a disability and/or End-Stage Renal Disease (ESRD) regardless of age. Medicare Part A (Hospital Insurance) provides coverage for services rendered in the hospital inpatient setting, Part B (Medical Insurance) provides coverage for services from doctors, outpatient medical care, and home health care, and Part D provides coverage for prescription drugs, including vaccinations. The Medicare Enrollment Database (EDB) and Common Medicare Environment (CME) contain information on Medicare beneficiaries' enrollment and entitlement. The American Community Survey (ACS) will provide information on beneficiaries' local population density, and the Area Deprivation Index (ADI) data from 2019 is used to assess ADI13 at the block group or at the zip-code level based on the last known address of the beneficiary in the EDB and the CME database.[6] ADI provides information on beneficiaries' local level of socioeconomic deprivation. Medicare institutional and professional claims data will be extracted from Shared Systems Data (SSD), a database updated daily with claims that have been enumerated but not yet adjudicated or paid. Vaccine exposure information used in this study will also be from SSD. Vaccination information will also be captured from Medicare Part D prescription drug claims contained in the

Prescription Drug Event (PDE) file. Medicare beneficiaries' skilled nursing facility and hospice residence status will be identified from SSD, and Medicare beneficiaries' nursing home residence status will be ascertained from the Minimum Data Set (MDS). MDS is a monthly-updated dataset with routine assessments during a beneficiary's stay at a Medicare- or Medicaid-certified nursing homes.

### 3.3 Study Period

The study period will be from Sunday, August 6, 2023, coinciding with the start of a typical flu season, to the Saturday with the most recently available data cut with 90% completeness, and represents the period across which we will be identifying RSV vaccinations and RSV-related outcomes of interest.

Beneficiary follow-up time will begin on the first date of high RSV circulation in a beneficiary's census tract or from the end of a prior RSV episode, whichever occurred later. Two high circulation rate definitions will be used:

1. An analysis will be conducted defining high circulation as 8 cases per 100,000 Medicare beneficiaries in a given census tract.
2. An analysis will be conducted considering all tracts to be in high-circulation after October 1, 2023.

### 3.4 Exposure and Index Date Definition

Exposure will be defined as the receipt of one dose of either RSV vaccine that has been approved by FDA (Pfizer RSVpreF or GSK RSVPreF3). Doses of GSK RSVPreF3 must be observed after May 3, 2023 and doses of Pfizer RSVpreF must observed after May 31, 2023, the dates in which FDA approved each vaccine in individuals aged ≥60 years. Medicare beneficiaries' vaccination status will be classified using Healthcare Common Procedure Coding System (HCPCS), Current Procedural Terminology (CPT), and National Drug Code (NDC) codes indicating RSV vaccination in any care setting.

Given that vaccine exposure status changes over time, the study will use a time-varying cohort approach that allows beneficiaries to contribute time to both cohorts. Specifically, a beneficiary will contribute time to the cohort that reflects their vaccine exposure status at each time point.

Prior to May 2023, no vaccination for RSV was approved in the United States. Additionally, CDC did not endorse the Advisory Committee on Immunization Practices (ACIP) recommendations regarding who could receive the vaccinations until June 29, 2023.[4] Unsurprisingly, observed RSV vaccination activity before the study start date, August 6, 2023, is

extremely low. As such, we excluded individuals with RSV vaccinations prior to the study start date.

Index date will be the study start date (August 6, 2023), where all individuals begin in the unvaccinated cohort. Vaccination status will be followed from this date, and beneficiaries will move from the unvaccinated to vaccinated cohort 14 days after vaccination.

### 3.5 Study Population

The primary study population will include Medicare older adults enrolled in Parts A/B Fee-for-Service (FFS) as well as Part D. Beneficiaries must meet the following criteria to be included in the study population:

- The beneficiary must be age 65 years or older on the index date August 6, 2023.
- The beneficiary must have been continuously enrolled in Medicare Parts A/B FFS and Part D for 365 days prior to index date and through the date follow-up begins, with no Part C (Medicare Advantage) enrollment at any time during this period.
- The beneficiary must not have evidence of chronic/regular dialysis in the 90 days prior to index date and through the date follow-up begins.
- The beneficiary must not be enrolled in hospice on index date.
- The beneficiary must not have an RSV episode starting between the index date and the date follow-up begins, nor an RSV episode starting before the index date and ending after their censor date.
- The beneficiary must not be residing in a nursing home on index date.
- The beneficiary must be alive on the date follow-up begins.
- The beneficiary have a valid address that can be mapped to a census tract
- The beneficiary must not have received a subsequent RSV vaccine after initial RSV vaccination.
- The beneficiary must not have an RSV vaccination before the index date.

### 3.6 Follow-up Time and Censoring

Beneficiaries are followed from either the first date of high circulation in their census tract after October 1, 2023, or from the end of a prior RSV episode, whichever occurred later. Individuals who received their vaccination prior to study start date will not be included in the study. Follow-up will continue until the occurrence of any of the following events:

- Discontinued from Medicare Parts A/B and/or D or enrollment into Part C
- Admission into a hospice care facility
- Admission into a nursing home facility
- Treatment of ESRD in a dialysis facility
- Occurrence of an outcome of interest

- Administration of a subsequent RSV vaccine after initial RSV vaccination
- Death
- End of the study period
- Consecutive periods of low circulation of RSV for the census tract

Individuals censored due to low RSV circulation may re-enter the study if their census tract returns to high RSV circulation following consecutive periods above a selected high circulation threshold.

### 3.7 Outcome Definitions

The primary outcome of interest will be RSV-related hospitalization, identified as inpatient claims using the following ICD-10 diagnosis codes:

**Table 2. ICD-10 Diagnosis Codes for RSV**

| Code | Description |
|---|---|
| B974 | Respiratory syncytial virus as the cause of diseases classified elsewhere |
| J121 | Respiratory syncytial virus pneumonia |
| J205 | Acute bronchitis due to respiratory syncytial virus |
| J210 | Acute bronchiolitis due to respiratory syncytial virus |

As patients with RSV could have multiple health care encounters during a single episode and reinfections can occur, distinct RSV infection episodes with consecutive RSV diagnoses will be constructed. Only one distinct RSV episode is allowed in the same RSV season and a distinct RSV episode must not have:

- a prior RSV episode that started within 180 days before the start of the current episode
- a prior RSV episode that ended within 60 days of the current RSV episode start date

Secondary outcomes of interest will be RSV-related deaths, severe inpatient complications, and RSV infections. These conditions will be defined and assessed in follow up analyses.

### 3.8 Covariate Summary

Covariates will be identified using data on beneficiary demographic or socioeconomic status characteristics from the CME at the time of the index date (August 6, 2023) or by the presence of administrative billing codes in the Medicare claims data. All medical conditions are flagged in

the 365 days prior to and including the index date, and includes prior influenza and/or COVID-19 vaccination status.[a]

### 3.8.1 *Time-Invariant Covariates*

Risk factors hypothesized to be associated with increased severe RSV-related outcomes will be included in the analysis, such as beneficiaries' demographics, socioeconomic status, general health status (including frailty, immunocompromised status, and presence of any specific chronic conditions), and flu and COVID-19 vaccination status. We will assess beneficiaries' demographic and socioeconomic characteristics using enrollment data, chronic conditions using ICD-10-CM diagnosis codes, and specific therapies and treatments using the ICD-10-PCS codes and HCPCS/CPT codes on inpatient and outpatient claims in the 365 days prior to and including index date.

### 3.8.2 *Time-Varying Covariates*

To account for the effects of local infection intensity on beneficiaries' risk of RSV-related outcomes, the local circulation rate will be summarized as rates varying every 14 days at the census tract-level (determined by beneficiaries' address information). More specifically, 14-day census tract-level infection rates will be calculated based on the number of Medicare beneficiaries who received any RSV diagnosis in any care setting (numerator) among all Medicare beneficiaries aged ≥65 years that are enrolled and living in a census tract in a 14-day period (denominator).

A Besag-York-Mollié (BYM) will be employed to enhance the precision of estimated infection rates by reducing noise, particularly for rates of smaller geographic units.[7] BYM modeling simultaneously incorporates spatial auto-correlation between regions and addresses unstructured regional differences to improve spatial smoothing.

A full list of covariates of interest and their data sources are included in Table 3 below.

**Table 3. List of Covariates**

| Covariates | Data Sources |
|---|---|
| **Age (Continuous)** | Medicare Enrollment Database |
| **Sex** | Medicare Enrollment Database |
| **CMS Medicare Race/Ethnicity** | Medicare Enrollment Database |
| **Dual Eligibility** | Medicare Enrollment Database |

[a] Prior influenza and/or COVID-19 vaccination must occur no earlier than the start of the 2022-2023 RSV season (August 7, 2022).

| Covariates | Data Sources |
|---|---|
| **Area Deprivation Index (ADI)** | ADI, Medicare Enrollment Database |
| **Reason for Entering Medicare** | Medicare Enrollment Database |
| **Frailty Conditions** | Medicare Claims |
| Frailty-Parkinson's Disease | Medicare Claims |
| Frailty-Cognitive Impairment | Medicare Claims |
| Frailty-Depression | Medicare Claims |
| Frailty-Paranoia | Medicare Claims |
| Frailty-Arthritis | Medicare Claims |
| Frailty-Chronic Skin Ulcer | Medicare Claims |
| Frailty-Skin and Soft Tissue Infection | Medicare Claims |
| Frailty-Pneumonia | Medicare Claims |
| Frailty-Mycoses | Medicare Claims |
| Frailty-Gout | Medicare Claims |
| Frailty-Falls | Medicare Claims |
| Frailty-Musculoskeletal Problems | Medicare Claims |
| Frailty-UTI | Medicare Claims |
| Frailty-Charlson Score>0 | Medicare Claims |
| Frailty-Hospital Admission in Past 6 Months | Medicare Claims |
| **Prior Vaccination Status** | |
| Influenza vaccinations | Medicare Claims |
| COVID-19 vaccinations | Medicare Claims |
| **Presence of Medical Conditions** | |
| Essential hypertension (without complications or other cardiovascular diseases) | Medicare Claims |
| Hypertension (with complications or other cardiovascular diseases) | Medicare Claims |
| Stroke/Transient ischemic attack | Medicare Claims |
| Chronic Kidney Disease | Medicare Claims |
| *Other cardiovascular diseases including:* | |
| Atrial fibrillation | Medicare Claims |
| Congestive heart failure | Medicare Claims |
| Coronary revascularization | Medicare Claims |
| Myocardial infarction | Medicare Claims |

| Covariates | Data Sources |
|---|---|
| Other cerebrovascular disease | Medicare Claims |
| Obesity | Medicare Claims |
| Chronic Obstructive Pulmonary Disease (COPD) | Medicare Claims |
| Chronic Kidney Disease | |
| *Other chronic respiratory diseases, including:* | |
| Asthma without COPD | Medicare Claims |
| Interstitial lung disease | Medicare Claims |
| Hypersensitivity pneumonitis | Medicare Claims |
| Bronchiectasis | Medicare Claims |
| Diabetes | Medicare Claims |
| Chronic liver disease | Medicare Claims |
| Neurological/Neurodevelopmental conditions | Medicare Claims |
| **Prior RSV Diagnosis** | Medicare Claims |
| **Immunocompromised Status** | Medicare Claims |
| **Time-Varying Census Tract-Level Circulation Rate** | Medicare Claims |
| **Population Density** | ACS, EDB/CME |

## 3.9 Statistical Analysis

For all analyses, we will use Marginal Structural Cox regression models to estimate the hazard ratios for RSV-related outcomes in the vaccinated cohort compared to the unvaccinated cohort. We will use Marginal Structural Models (MSM) to address our concerns that several covariates of interest are not balanced across treatment stages in our study.[8,9] Specifically, the average treatment effect in the treated population (ATT) was estimated using inverse probability weighting (IPW) to adjust for factors potentially affecting vaccination status and RSV risk among individuals. Relative effectiveness is calculated as (1−HR) × 100%, where HR is the estimated hazard ratio between the cohorts.

### 3.9.1 *Marginal Structural Models*

MSMs aim to balance potential confounders across treatment stages in a study. Confounding adjustment is implemented by propensity score weighting. Specifically, MSMs assign a weight to each observation based on the inverse of their likelihood of receiving a particular treatment regimen. These weights are employed in the models to create a pseudo-population to analytically isolate the effect of the treatment on the outcome of interest.

### 3.9.2 *Descriptive Analyses*

We will produce descriptive statistics to summarize each of the following:

- Impact of various inclusion/exclusion criteria on the population of interest
- Uptake of vaccines by brand
- Geographical distribution of vaccinations (e.g., across HHS regions)
- Covariate balance

### 3.9.3 *Covariate Balancing Method*

To achieve balance between cohorts, we will use IPW with ATT weights. Because we are using an MSM framework to follow all individuals initially as unvaccinated, we will use one time-varying treatment weight and one time-varying censoring weight to calculate the final inverse probability weight (at 7-day intervals or when a beneficiary switches cohorts). [10]

Covariate balance across cohorts for all analyses will be evaluated using the standardized mean difference (SMD) of all covariates over time. SMD is defined as the difference in means (for continuous variables) or proportions (for categorical/binary variables) of a variable divided by the pooled standard deviation of the variable.[11,12] A standardized mean difference of 0.1 or less indicates a negligible difference between groups.

### 3.9.4 *Primary Analysis*

To compare the effectiveness of each vaccine, we will use multiple models. These models will be fit, as power allows, using multiple Cox-MSMs including main effects and/or interaction.

The primary model will not assess waning, and the secondary follow up models will incorporate separate assumptions: one in which waning is the same for each brand, and one in which waning varies by brand. Likelihood ratio tests will be used to measure the model performance of the primary and secondary models.

The effects of brand-specific waning immunity on the effectiveness will be modeled at discrete time intervals based on clinical knowledge and follow-up time distribution. When appropriate, the predicted VEs and their CIs will be plotted. To reduce the impact of residual confounding on VE estimates, doubly robust results will be reported with propensity score model covariates included in the outcome model. The statistical significance of the estimates will be determined by two-sided hypothesis tests where the alternative hypotheses are non-zero effects.

**Primary Model**

$$\lambda(t|x) = \lambda_0(\mathrm{t}) * \exp(\beta_1 * Pfizer(t) + \beta_2 * GSK(t) + \sum \theta_j * u_j + \sum \gamma_k * v_k(t))$$

where

$t$ = time since index date

$\lambda_0$ = baseline hazard
$Pfizer$ = 1 where a beneficiary has received a Pfizer vaccine and 0 otherwise
$GSK$ = 1 where a beneficiary has received a GSK vaccine and 0 otherwise
$u_j$= jth time-fixed covariate and $\theta_j$ = corresponding coefficient
$v_k(t)$= kth time-varying covariate and $\gamma_k$ = corresponding coefficient

$$VE_{Pfizer} = (1 - \exp(\beta_1)) * 100\%$$
$$VE_{GSK} = (1 - \exp(\beta_2)) * 100\%$$
$$VE_{Combined} = (1 - \exp(w_1 * \beta_1 + w_2 * \beta_2)) * 100\%$$
$$RVE_{Pfizer\ vs\ GSK} = (1 - \exp(\beta_1 - \beta_2)) * 100\%$$

where $w_1$ and $w_2$ are weights for Pfizer and GSK, respectively, corresponding to the proportion of vaccinated individuals with that vaccine such that $w_1 + w_2 = 1$

### 3.9.5 *Secondary Analyses*

**Secondary Model – Waning Immunity**

$$\lambda(t|x) = \lambda_0(t) * \exp(\beta_1 * Pfizer(t) + \beta_2 * GSK(t) + \sum \theta_j * u_j + \sum \gamma_k * v_k(t) + g_1(Pfizer(t), TimeSinceVaccination(t)) + g_2(GSK(t), TimeSinceVaccination(t)))$$

where

$g_1(Pfizer, TimeSinceVaccination)$ = function interacting Pfizer vaccinated status and time since Pfizer vaccination

$g_2(GSK, TimeSinceDose)$ = function interacting GSK vaccinated status and time since GSK vaccination

$$VE_{Pfizer} = (1 - \exp(\beta_1 + g_1(TimeSinceVaccination))) * 100\%$$
$$VE_{GSK} = (1 - \exp(\beta_2 + g_2(TimeSinceVaccination))) * 100\%$$
$$VE_{Combined} = (1 - \exp(w_1 * (\beta_1 + g_1(TimeSinceVaccination)) + w_2 * (\beta_2 + g_2(TimeSinceVaccination)))) * 100\%$$
$$RVE_{Pfizer\ vs\ GSK} = (1 - \exp(\beta_1 - \beta_2 + g_1(TimeSinceVaccination) - g_2(TimeSinceVaccination))) * 100\%$$

### 3.9.6 *Follow Up Analyses*

The protocol focuses on specifications for the primary analysis (absolute vaccine effectiveness) of the primary outcome (RSV-related hospitalizations) in the primary population (community-dwelling Medicare beneficiaries without ESRD). Following completion of this analysis, follow up analyses may be pursued, including subgroup and sensitivity analyses on the current population of interest as well as secondary outcomes and alternate populations (nursing home population).

#### *3.9.6.1 Sensitivity Analyses*

Sensitivity analyses may include:

- Sensitivity analyses will be conducted to determine the impact of performing analyses in which periods of high circulation within census tracts have alternative thresholds than the high circulation threshold used in the main analysis. This includes adopting no requirement for high circulation for beneficiaries to be included.
- Sensitivity analyses to explore alternative definition of distinct RSV episodes.
- A sensitivity analysis not allowing cohort switching may be conducted.
- The use of a negative control outcome.

#### *3.9.6.2 Subgroup Analyses*

If sample size permits, we may conduct subgroup analyses based on covariates of interest, including but not limited to:

- Age categories
- Immunocompromised status
- At least one high risk medical conditions of interest
- Frailty conditions

#### *3.9.6.3 Secondary Outcomes*

The analysis may be conducted on a set of secondary outcomes, including RSV-related mortality, RSV infection, and severe complications of RSV hospitalizations (e.g., ICU/CCU, ventilation, and inpatient mortality). Power for these outcomes will be assessed before proceeding with the analysis.

#### *3.9.6.4 Nursing Home Population*

The analysis may be repeated in the nursing home population following assessment of power. In the event that further analyses are deemed necessary by the study team, protocol addenda may be produced outlining additional study specifications.

## 4. ACKNOWLEDGEMENT AND CERTIFICATION

### Electronic Signature

By submitting an electronic signature under this Acknowledgement and Certification, you are acknowledging that you have read and agree to the contents of this protocol. By signing below, you indicate your approval for the analyses described in this protocol.

X Yun Lu -S

Digitally signed by Yun Lu -S
Date: 2024.05.22 21:20:22 -04'00'

Yun Lu
Mathematical Statistician, FDA/CBER/OBPV/DABRA

X Richard A. Forshee -S

Digitally signed by Richard A. Forshee -S
Date: 2024.05.23 07:24:45 -04'00'

Richard A Forshee
Office Deputy Director, FDA/CBER/OBPV

X Michael Wernecke

Digitally signed by Michael Wernecke
Date: 2024.05.22 15:55:45 -07'00'

Michael Wernecke
Research Manager, Acumen, LLC

## 5. REFERENCES


1. Administration USFD. FDA Approves First Respiratory Syncutial Virus (RSV) Vaccine: Arexvy Approved for Individuals 60 Years of Age and Older. 2023; https://www.fda.gov/news-events/press-announcements/fda-approves-first-respiratory-syncytial-virus-rsv-vaccine, 2024.
2. Prevention CfDCa. Respiratory Syncytial Virus (RSV). 2024; https://www.cdc.gov/rsv/index.html, 2024.
3. Administration USFD. ABRYSVO. 2024; https://www.fda.gov/vaccines-blood-biologics/abrysvo, 2024.
4. Prevention CfDCa. CDC Recommends RSV Vaccine For Older Adults. 2023; https://www.cdc.gov/media/releases/2023/s0629-rsv.html, 2024.
5. Robins JM. Marginal Structural Models. Paper presented at: American Statistical Association, Section on Bayesian Statistical Sciences1997.
6. Kind AJ, Buckingham WR. Making Neighborhood-Disadvantage Metrics Accessible - The Neighborhood Atlas. *New England Journal of Medicine.* 2019;378(26):2456-2458.
7. Besag J, York J, Mollié A. Bayesian image restoration, with two applications in spatial statistics. *Annals of the Institute of Statistical Mathematics.* 1991;43(1):1-20.
8. Robins JM, Hernan MA, Brumback B. Marginal structural models and causal inference in epidemiology. *Epidemiology.* 2000;11(5):550-560.
9. Hernan MA, Brumback B, Robins JM. Marginal structural models to estimate the causal effect of zidovudine on the survival of HIV-positive men. *Epidemiology.* 2000;11(5):561-570.
10. Benjamini Y, Hochberg Y. Controlling the False Discovery Rate: A Practical and Powerful Approach to Multiple Testing. *Royal Statistical Society.* 1995;57(1):289-300.
11. Austin PC. Balance diagnostics for comparing the distribution of baseline covariates between treatment groups in propensity-score matched samples. *Stat Med.* 2009;28(25):3083-3107.
12. Mamdani M, Sykora K, Li P, et al. Reader's guide to critical appraisal of cohort studies: 2. Assessing potential for confounding. *BMJ.* 2005;330(7497):960-962.
13. Greenberg JA, Hohmann SF, Hall JB, Kress JP, David MZ. Validation of a Method to Identify Immunocompromised Patients with Severe Sepsis in Administrative Databases. *Ann Am Thorac Soc.* 2016;13(2):253-258.
14. Poutsiaka DD, Davidson LE, Kahn KL, Bates DW, Snydman DR, Hibberd PL. Risk factors for death after sepsis in patients immunosuppressed before the onset of sepsis. *Scand J Infect Dis.* 2009;41(6-7):469-479.
15. Tolsma V, Schwebel C, Azoulay E, et al. Sepsis severe or septic shock: outcome according to immune status and immunodeficiency profile. *Chest.* 2014;146(5):1205-1213.
16. Rubin LG, Levin MJ, Ljungman P, et al. 2013 IDSA clinical practice guideline for vaccination of the immunocompromised host. *Clin Infect Dis.* 2014;58(3):e44-100.
17. van der Wal WM, Noordzij M, Dekker FW, et al. Comparing mortality in renal patients on hemodialysis versus peritoneal dialysis using a marginal structural model. *The International Journal of Biostatistics.* 2010;6(1).
18. Hernán MA, Hernández-Díaz S, Robins JM. A structural approach to selection bias. *Epidemiology.* 2004;15(5):615-625.
19. Cole SR, Hernán MA. Constructing inverse probability weights for marginal structural models. *Am J Epidemiol.* 2008;168(6):656-664.

# 6. APPENDIX

## A. Immunocompromised Algorithm

Immunocompromised beneficiaries will be identified using a modified version of the algorithm developed and validated by Greenberg et al. (2015).[13] Their algorithm reported a positive predictive value (PPV) of 94.4% (95% CI 88.8–97.7), negative predictive value (NPV) of 94.3% (95% 91.0-96.6), sensitivity of 87.4% (95% CI 80.6–92.5%) and specificity of 97.6% (95% CI 95.0–99.9%). The Greenberg algorithm was used to inform the development of a previous FDA algorithm for an immunocompromised population. However, the Greenberg algorithm used only ICD-9-CM codes, so these have been mapped to ICD-10-CM. The algorithm also includes some additional codes from the Agency for Healthcare Research and Quality (AHRQ) criteria for identifying individuals at risk of immunosuppression. This approach provides a comprehensive algorithm for immunocompromised individuals that builds on an independently validated approach using the clinical expertise of the CBER team.

A key challenge in developing this algorithm was the lack of a standard, universal definition for 'clinical immunosuppression.' As a result, the authors used a previously-published, independently-validated algorithm presented by Greenberg and colleagues. In this study, authors developed seven categories of immunosuppression, based on previously published classification schemes.[14-16] A modified version of this classification scheme, which we will use, has eight mutually exclusive (i.e., implemented one at a time) categories:

1. HIV/AIDS
2. Hematological Malignancy and Related Conditions
3. Immune Deficiencies (treatment-dependent)
4. Immune Deficiencies (treatment-independent)
5. Solid Malignancy
6. Transplant and Related Conditions
7. Rheumatological/Inflammatory
8. Dialysis

The current immunocompromised algorithm searches the 6 months prior to index date.

## B. RSV Vaccine Codes

Appendix Table 1. Vaccine Codes for Approved Respiratory Syncytial Virus (RSV) Vaccines

| Scientific Name | Proprietary Name and Manufacturer | Vaccine Authorization Date | CVX Code | NDC 11 Labeler Product ID | HCPCS / CPT Code |
|---|---|---|---|---|---|
| RSVPreF3 | AREXVY – GlaxoSmithKline Biologics | May 3, 2023 | 303 | 58160072303<br>58160074403<br>58160084811 | 90679 |
| RSVpreF | ABRYSVO – Pfizer Inc. | May 31, 2023 | 305 | 00069020701<br>00069025001<br>00069034401<br>00069034405<br>00069034410 | 90678 |

## C. Marginal Structural Models Statistical Implementation

### a. Data Preparation

For the weighting model we used the following data structure. For each individual *i*, the data matrix contained rows $j = 0, \ldots, n_i - 1$, which corresponded to time points from baseline up to the individual end time, spaced 7 days apart since index date. Subjects were represented by the set $\{A_{ij}, Y_{ij}, Z_{ij}, L_{ij}, V_i\}$.

We indicated the RSV vaccination for each individual *i* with $A_{ij}$, which is a categorical treatment variable with *g* categories (1 = untreated, 2 = Pfizer, 3 = GSK). We indicated the observed outcome status at time point *j* with $Y_{ij}$ (0 = non-case, 1 = case), and the event of dropout at time point *j* with $Z_{ij}$ (0 = dropout, 1 = no-dropout). The most recent time-varying covariate measurements taken before time point *j* were contained in $L_{ij}$. At time point $j$, $L_{ij}$ was measured before $A_{ij}$. The baseline covariates were contained in $V_i$, which is time-independent and thus is fixed over time.

The individual end time is determined as the minimum of the time of outcome and the time of censoring events. Individuals are right-censored because we are not able to observe outcomes due to, for example, death, disenrollment, and the end of the follow-up.

### b. IPW adjustment for the effect of baseline and time-varying covariates on time-varying first vaccination

To adjust for the effect of the baseline time-fixed covariates $V_i$ and time-varying covariates $L_{ij}$ on the choice of initiating RSV vaccination, $A_{ij}$, we used IPW similar to its use in van der Wal et al. (2010).[17] We estimated the ATT weights where the treated group was any vaccinated individual:

$$sw_{ij}^{0} = \prod_{k=1}^{j} \frac{\sum_{m=2}^{g} P\left[A_{ik} = m \middle| \bar{L}_{ik} = \bar{l}_{ik}, V_i = v_i\right]}{P\left[A_{ik} = a_{ik} \middle| \bar{L}_{ik} = \bar{l}_{ik}, V_i = v_i\right]} \times \frac{P[A_{ik} = a_{ik}]}{\sum_{m=2}^{g} P[A_{ik} = m]} \tag{1}$$

The first term of (1) determines the unstabilized ATT weight, and the second term produces stabilized weights. The denominator of the first term contains the probability of receiving a vaccination $a_{ik}$ given an individual's observed baseline and time-varying covariate values $l_{ik}$ and $v_i$. Weighting individuals *i* by $w_i = 1/P[A_{ik} = a_{ik} \mid L_{ik} = l_{ik}, V_i = v_i]$ would result in adjusting for the effect of baseline and time-varying covariates on receiving a vaccination. The numerator of the first term results in ATT-vaccinated weights and contains the probability of receiving either the Pfizer or GSK vaccine given given an individual's observed baseline and time-varying covariate values $l_{ik}$ and $v_i$. The probability of receiving vaccine *h* is estimated using a pooled multinomial logistic regression model:

$$P\left[A_{ik} = h \middle| \bar{L}_{ik} = \bar{l}_{ik}, V_i = v_i\right] = \begin{cases} \dfrac{1}{1 + \sum_{m=2}^{g} \exp(\alpha_k + \beta_m \, \bar{L}_{ik} + \gamma_m \, V_i)}, & h = 1 \\ \dfrac{\exp(\beta_h \, \bar{L}_{ik} + \gamma_h V)}{1 + \sum_{m=2}^{g} \exp(\alpha_k + \beta_m \, \bar{L}_{ik} + \gamma_m \, V_i)}, & h > 1 \end{cases} \quad (2)$$

We assumed that the effect of the time-varying covariate history $\bar{L}_{ij}$ was fully expressed via the last value $L_{ij}$.

A propensity score close to 0 for certain subjects may result in extremely large weights. Thus, we stabilize these weights in (1), increasing efficiency. [5] The numerator of the stabilization term contains the marginal probability of being in an exposure category (*h*th exposure category) at each time point *k* for subject $i_k$, which is simply the proportion of subjects who are in the exposure category *h* at time point *i*. The denominator contains the marginal probability of reciving any vaccine does at each time point *k* for subject $i_k$.

### c. IPW adjustment for informative censoring due to dropout / loss of follow-up

Similar to the correction for informative censoring used in Hernan et al. (2004),[18] we adjusted for the effect of baseline and time-varying covariates as well as treatment status on the probability of dropout, by weighting the measurements, indexed by *ij*, by the stabilized weights

$$sw_{ij}^{\star} = \prod_{k=1}^{j} \frac{P[Z_{ik} = z_{ik}]}{P\left[Z_{ik} = z_{ik} \middle| A_{ik} = a_{ik}, \bar{L}_{ik} = \bar{l}_{ik}, V_i = v_i\right]} \quad (3)$$

The factors in the numerator of (3) contain the probability of the dropout status at each time point, $z_{ik}$. The factors in the denominator (3) contain the probability of the dropout status at each time point, given the time-varying treatment history, the observed time-varying covariate history up to the present time point and observed baseline covariates. These factors were estimated analogously to the estimation of the factors in (1), using a pooled logistic regression model.

$$P\left[Z_{ik}=z_{ik}\middle|A_{ik}=a_{ik},\bar{L}_{ik}=\bar{l}_{ik},V_i=v_i\right] = \frac{\exp(\alpha_k+\beta_1 A_{ik}+\beta_2\,\bar{L}_{ik}+\beta_3 V_i)}{1+\exp(\alpha_k+\beta_1 A_{ik}+\beta_2\,\bar{L}_{ik}+\beta_3 V_i)} \quad (4)$$

We estimated that the effects of the time-varying covariate history $\bar{L}_{ij}$ was fully expressed via the last value $L_{ij}$.

### d. Construction of exposure allocation models

The models used to estimate the denominators of the weights $sw_{ij}^{0}$ and $sw_{ij}^{\star}$ as described in the previous sections, were constructed as follows. All individuals entered the study on August 6, 2023, the start of the RSV season, which is their index date and defined as time 0. We then use model (1) to calculate vaccine dose treatment weight $sw_{ij}^{0}$ from baseline to the end of the study, and model (3) to calculate dropout weight $sw_{ij}^{\star}$ from baseline to the end of the study.

### e. Outcome model and inference

The absolute VE outcome models were fitted with elements $ij$ weighted by the product $sw_{ij}^{0}\times sw_{ij}^{\star}$, using the vaccine dose treatment weights and censoring weights. The weighting removed the effect of baseline and time-varying covariates on the treatment status of receiving a vaccine dose, and the effect of baseline and time-varying covariates on the probability of dropout. We assumed that there was no additional unmeasured confounding and informative censoring, that (2) and (4) had been correctly specified and that the population values of the probabilities estimated in the denominator of (1) and (3) were always larger than 0, which is the assumption of experimental treatment assignment (ETA).[19]
