# Supplementary material for: Respiratory Syncytial Virus Vaccine Effectiveness Among US Older Adults, 2023-2024 Season
Source: JAMA Netw Open. 2026 Sep 18;9(9):e2634788. doi: 10.1001/jamanetworkopen.2026.34788 (PMC13589284; doi:10.1001/jamanetworkopen.2026.34788)
Supplement: Supplementary file 2 [file jamanetwopen-e2634788-s002.pdf]

Supplementary Online Content

Lu Y, Smith ER, McEvoy R, et al. Respiratory syncytial virus vaccine effectiveness among US older adults, 2023-2024 season. *JAMA Netw Open.* 2026;9(9):e2634788. doi:10.1001/jamanetworkopen.2026.34788

eMethods 1. Population Construction

eTable 1. List of Covariates

eMethods 2. Statistical Analyses

eTable 2. Vaccine Codes for Approved Respiratory Syncytial Virus (RSV) Vaccines

eTable 3. Censoring Reason Summary

eTable 4. Distribution of Follow Up Time Among Study Participants, Days

eTable 5. Specifications for Age Investigation

eTable 6. VE Estimates for Best Fitting Model With Age-Interaction Term: 2-Interior Knot Age Spline and Linear Age Interaction Term

eFigure. VE Estimates for Best Fitting Model With Age-Interaction Term: 2-Interior Knot Age Spline and Linear Age Interaction Term

eTable 7. VE Results by Immunocompromised Subcategories

This supplementary material has been provided by the authors to give readers additional information about their work.

© 2026 Lu Y et al. *JAMA Network Open.*

S1. Supplemental Methods

eMethods 1. Population Construction

The primary study population includes Medicare older adults enrolled in Parts A/B Fee-for-Service (FFS) as well as Part D. Beneficiaries must meet the following criteria to be included in the study population:

- The beneficiary must be age 65 years or older on the index date August 6, 2023.
- The beneficiary must have been continuously enrolled in Medicare Parts A/B FFS and Part D for 365 days prior to index date and through the date follow-up begins, with no Part C (Medicare Advantage) enrollment at any time during this period.
- The beneficiary must not have evidence of chronic/regular dialysis in the 90 days prior to index date and through the date follow-up begins.
- The beneficiary must not be enrolled in hospice on index date.
- The beneficiary must not have an RSV episode starting between the index date and the date follow-up begins, nor an RSV episode starting before the index date and ending after their censor date.
- The beneficiary must not be residing in a nursing home on index date.
- The beneficiary must be alive on the date follow-up begins.
- The beneficiary must have a valid address that can be mapped to a census tract
- The beneficiary must not have received a subsequent RSV vaccine after initial RSV vaccination.
- The beneficiary must not have an RSV vaccination before the index date.

Follow-Up Time and Censoring

Beneficiaries are followed from either the first date of high circulation in their census tract after October 1, 2023, or from the end of a prior RSV episode, whichever occurred later. Beneficiaries who were in the midst of an RSV episode at study start were not at risk by definition, therefore we delayed their follow up begin date until after their RSV episode ended and they were at risk again. Individuals who received their vaccination prior to study start date will not be included in the study. Follow-up will continue until the occurrence of any of the following events:

- Discontinued from Medicare Parts A/B and/or D or enrollment into Part C
- Admission into a hospice care facility
- Admission into a nursing home facility
- Treatment of ESRD in a dialysis facility
- Occurrence of an outcome of interest
- Administration of a subsequent RSV vaccine after initial RSV vaccination
- Death

- End of the study period
- Consecutive periods of low circulation of RSV for the census tract

Individuals censored due to low RSV circulation may re-enter the study if their census tract returns to high RSV circulation following consecutive periods above a selected high circulation threshold.

RSV Circulation Calculation

Census tract level RSV circulation is used to determine if a beneficiary is in a "high-circulation" tract and as a time-varying covariate. 14-day census tract-level infection rates will be calculated based on the number of Medicare beneficiaries who received any RSV diagnosis in any care setting (numerator) among all Medicare beneficiaries aged ≥65 years that are enrolled and living in a census tract in a 14-day period (denominator). A Besag-York-Mollié (BYM) model will be employed to enhance the precision of estimated infection rates by reducing noise, particularly for rates of smaller geographic units.[1] BYM modeling simultaneously incorporates spatial auto-correlation between regions and addresses unstructured regional differences to improve spatial smoothing.

Covariates

Covariates are identified using data on beneficiary demographic or socioeconomic status characteristics from the CME at the time of the index date (August 6, 2023) or by the presence of administrative billing codes in the Medicare claims data. All medical conditions are flagged in the 365 days prior to and including the index date, and includes prior influenza and/or COVID-19 vaccination status occurring no earlier than the start of the 2022-2023 RSV season (August 7, 2022). Covariates were selected based on their potentiality to be a confounder and/or their hypothesized association with the outcome of interest. Broad categories of covariates included: demographic characteristics (age, sex, race/ethnicity), indicators of socio-economic status (Medicare/Medicaid dual eligibility status), general health conditions, frailty indicators, and prior vaccination. We also included geographic-level indicators such as ADI, census-tract-level population density and time-varying census-tract-level RSV circulation as measures of potential contagion

Time-fixed covariates include risk factors hypothesized to be associated with increased severe RSV-related outcomes, such as beneficiaries' demographics, socioeconomic status, general health status (including frailty, immunocompromised status, and presence of any specific chronic conditions), and flu and COVID-19 vaccination status. We will assess beneficiaries' demographic and socioeconomic characteristics using enrollment data, chronic conditions using ICD-10-CM diagnosis codes, and specific therapies and treatments using the ICD-10-PCS codes and HCPCS/CPT codes on inpatient and outpatient claims in the 365 days prior to and including index date. To account for the effects of local infection intensity on beneficiaries' risk of RSV-

---

[1] Besag J, York J, Mollié A. Bayesian image restoration, with two applications in spatial statistics. *Annals of the Institute of Statistical Mathematics.* 1991;43(1):1-20.

© 2026 Lu Y et al. *JAMA Network Open.*

related outcomes, the census tract level RSV circulation rate is included as a time-varying covariate.

The full list of covariates and their data source is included below:

eTable 1. List of Covariates

Covariates	Data Sources
**Age (Continuous)**	Medicare Enrollment Database
**Sex**	Medicare Enrollment Database
**CMS Medicare Race/Ethnicity**	Medicare Enrollment Database
**Medicare/Medicaid Dual Eligibility**	Medicare Enrollment Database
**Area Deprivation Index (ADI)**	ADI, Medicare Enrollment Database
**Reason for Entering Medicare**	Medicare Enrollment Database
**Frailty Conditions**	Medicare Claims
Frailty-Parkinson's Disease	Medicare Claims
Frailty-Cognitive Impairment	Medicare Claims
Frailty-Depression	Medicare Claims
Frailty-Paranoia	Medicare Claims
Frailty-Arthritis	Medicare Claims
Frailty-Chronic Skin Ulcer	Medicare Claims
Frailty-Skin and Soft Tissue Infection	Medicare Claims
Frailty-Pneumonia	Medicare Claims
Frailty-Mycoses	Medicare Claims
Frailty-Gout	Medicare Claims
Frailty-Falls	Medicare Claims
Frailty-Musculoskeletal Problems	Medicare Claims
Frailty-UTI	Medicare Claims
Frailty-Charlson Score>0	Medicare Claims
Frailty-Hospital Admission in Past 6 Months	Medicare Claims
**Prior Vaccination Status**	
Influenza vaccinations	Medicare Claims
COVID-19 vaccinations	Medicare Claims
**Presence of Medical Conditions**	
Essential hypertension (without complications or other cardiovascular diseases)	Medicare Claims
Hypertension (with complications or other cardiovascular diseases)	Medicare Claims
Stroke/Transient ischemic attack	Medicare Claims
Chronic Kidney Disease	Medicare Claims
Other cardiovascular diseases including:	
Atrial fibrillation	Medicare Claims
Congestive heart failure	Medicare Claims
Coronary revascularization	Medicare Claims
Myocardial infarction	Medicare Claims
Other cerebrovascular disease	Medicare Claims
Obesity	Medicare Claims

© 2026 Lu Y et al. *JAMA Network Open.*

Covariates	Data Sources
Chronic Obstructive Pulmonary Disease (COPD)	Medicare Claims
Chronic Kidney Disease	
Other chronic respiratory diseases, including:	
Asthma without COPD	Medicare Claims
Interstitial lung disease	Medicare Claims
Hypersensitivity pneumonitis	Medicare Claims
Bronchiectasis	Medicare Claims
Diabetes	Medicare Claims
Chronic liver disease	Medicare Claims
Neurological/Neurodevelopmental conditions	Medicare Claims
**Prior RSV Diagnosis**	Medicare Claims
**Immunocompromised Status**	Medicare Claims
**Time-Varying Census Tract-Level Circulation Rate**	Medicare Claims
**Population Density**	ACS, EDB/CME

Immunocompromised Status: In this study, authors developed seven categories of immunosuppression, based on previously published classification schemes.[2] A modified version of this classification scheme is used and has eight categories:

1. HIV/AIDS
2. Hematological Malignancy and Related Conditions
3. Immune Deficiencies (treatment-dependent)
4. Immune Deficiencies (treatment-independent)
5. Solid Malignancy
6. Transplant and Related Conditions
7. Rheumatological/Inflammatory
8. Dialysis

The current immunocompromised algorithm searches the 12 months prior to index date.

[2] Poutsiaka DD, Davidson LE, Kahn KL, Bates DW, Snydman DR, Hibberd PL. Risk factors for death after sepsis in patients immunosuppressed before the onset of sepsis. *Scand J Infect Dis.* 2009;41(6-7):469-479.
Tolsma V, Schwebel C, Azoulay E, et al. Sepsis severe or septic shock: outcome according to immune status and immunodeficiency profile. *Chest.* 2014;146(5):1205-1213.
Rubin LG, Levin MJ, Ljungman P, et al. 2013 IDSA clinical practice guideline for vaccination of the immunocompromised host. *Clin Infect Dis.* 2014;58(3):e44-100.

© 2026 Lu Y et al. *JAMA Network Open.*

eMethods 2. Statistical Analyses

For all analyses, we will use Marginal Structural Poisson regression models to estimate the hazard ratios for RSV-related outcomes in the vaccinated cohort compared to the unvaccinated cohort. We will use Marginal Structural Models (MSM) to address time-varying exposures.[3] Specifically, the average treatment effect (ATE) will be estimated using inverse probability weighting (IPW) to adjust for factors potentially affecting vaccination status and RSV risk among individuals. Relative effectiveness is calculated as (1−HR) × 100%, where HR is the estimated hazard ratio between the cohorts.

To achieve balance between cohorts, we will use IPW with ATE weights. Because we are using an MSM framework to follow all individuals initially as unvaccinated, we will use one time-varying treatment weight and one time-varying censoring weight to calculate the final inverse probability weight (at 7-day intervals or when a beneficiary switches cohorts).[4]

Covariate balance across cohorts for all analyses will be evaluated using the standardized mean difference (SMD) of all covariates over time. SMD is defined as the difference in means (for continuous variables) or proportions (for categorical/binary variables) of a variable divided by the pooled standard deviation of the variable.[5] A standardized mean difference of 0.1 or less indicates a negligible difference between groups.

Primary Model

$$\lambda(t|x) = \lambda_0(t) * \exp(\beta_1 * Pfizer(t) + \beta_2 * GSK(t) + \sum \theta_j * u_j + \sum \gamma_k * v_k(t))$$

where

t = time since index date
λ_0 = baseline hazard
$Pfizer$ = 1 where a beneficiary has received a Pfizer vaccine and 0 otherwise
GSK = 1 where a beneficiary has received a GSK vaccine and 0 otherwise
u_j= jth time-fixed covariate and θ_j = corresponding coefficient
$v_k(t)$= kth time-varying covariate and γ_k = corresponding coefficient

$$VE_{Pfizer} = (1 - \exp(\beta_1)) * 100\%$$
$$VE_{GSK} = (1 - \exp(\beta_2)) * 100\%$$
$$VE_{Combined} = (1 - \exp(w_1 * \beta_1 + w_2 * \beta_2)) * 100\%$$

---

[3] Robins JM, Hernan MA, Brumback B. Marginal structural models and causal inference in epidemiology. *Epidemiology.* 2000;11(5):550-560.
Hernan MA, Brumback B, Robins JM. Marginal structural models to estimate the causal effect of zidovudine on the survival of HIV-positive men. *Epidemiology.* 2000;11(5):561-570
[4] Benjamini Y, Hochberg Y. Controlling the False Discovery Rate: A Practical and Powerful Approach to Multiple Testing. *Royal Statistical Society.* 1995;57(1):289-300.
[5] Austin PC. Balance diagnostics for comparing the distribution of baseline covariates between treatment groups in propensity-score matched samples. *Stat Med.* 2009;28(25):3083-3107.
Mamdani M, Sykora K, Li P, et al. Reader's guide to critical appraisal of cohort studies: 2. Assessing potential for confounding. *BMJ.* 2005;330(7497):960-962.

where w_1 and w_2 are weights for Pfizer and GSK, respectively, corresponding to the proportion of vaccinated individuals with that vaccine such that $w_1 + w_2 = 1$

© 2026 Lu Y et al. *JAMA Network Open.*

S2. Exposure Definition

eTable 2. Vaccine Codes for Approved Respiratory Syncytial Virus (RSV) Vaccines

Scientific Name	Proprietary Name and Manufacturer	Vaccine Authorization Date	CVX Code	NDC 11 Labeler Product ID	HCPCS / CPT Code
RSVPreF3	AREXVY – GlaxoSmithKline Biologics	May 3, 2023	303	58160072303<br>58160074403<br>58160084811	90679
RSVpreF	ABRYSVO – Pfizer Inc.	May 31, 2023	305	00069020701<br>00069025001<br>00069034401<br>00069034405<br>00069034410	90678

© 2026 Lu Y et al. *JAMA Network Open.*

S3. Follow Up Time and Censoring

eTable 3. Censoring Reason Summary

Censoring Reason	# of Patients	% of Patients
Discontinued from Medicare Parts A/B and/or D or enrollment into Part C	646,921	4.37%
Admission into a hospice care facility	72,408	0.49%
Admission into a nursing home facility	66,850	0.45%
Treatment of ESRD in a dialysis facility	3,589	0.02%
Occurrence of an outcome of interest (RSV-related hospitalization)	13,055	0.09%
Administration of a subsequent RSV vaccine after initial RSV vaccination	5,029	0.03%
Death	60,148	0.41%
End of the study period	13,838,114	93.38%
Consecutive periods of low circulation of RSV for the census tract	90,013	0.61%
Multiple	23,099	0.16%

eTable 4. Distribution of Follow Up Time Among Study Participants, Days

Follow-Up Time Distribution (Days)						
Mean	Standard Deviation	Minimum	25th Percentile	50th Percentile	75th Percentile	Maximum
123.88	23.41	1	112	126	140	154

© 2026 Lu Y et al. *JAMA Network Open.*

S4. Effect Modification by Age Investigation

To assess potential effect modification by age, we compared models including an interaction term for age and RSV vaccination, with both the base age term and the interaction term modeled non-linearly. We compared five models with the following specifications. All splines are natural splines with boundary knots at the 2.5% and 97.5% percentiles of data, and internal knots at 1/3 and 2/3 of the data.

eTable 5. Specifications for Age Investigation

Model Number	Base Age Term	Interacted Age * VE Term
1	Linear	Linear
2	1-knot spline	1-knot spline
3	2-knot spline	2-knot spline
4	2-knot spline	Linear
5	2-knot spline	1-knot spline

© 2026 Lu Y et al. *JAMA Network Open.*

Model #4, with the 2-knot spline for age and linear interaction term had the lowest AIC and was selected. This model did not find evidence of effect modification by age, as the interaction term was not significant. VE predictions by age for this model are shown below:

eTable 6. VE Estimates for Best Fitting Model With Age-Interaction Term: 2-Interior Knot Age Spline and Linear Age Interaction Term

**Age Prediction**	**Overall VE**	**Brand-Specific VE**	
	**RSV Vaccinated vs. Unvaccinated**	**Pfizer vs. Unvaccinated**	**GSK vs. Unvaccinated**
Age 66	80.9% (77.2%, 84.0%)	81.6% (74.4%, 86.7%)	80.6% (76.2%, 84.2%)
Age 75	81.3% (79.2%, 83.1%)	84.4% (81.1%, 87.2%)	79.5% (76.9%, 81.9%)
Age 85	81.6% (79.4%, 83.6%)	87.1% (83.6%, 89.8%)	78.3% (75.4%, 80.9%)

© 2026 Lu Y et al. *JAMA Network Open.*

eFigure. VE Estimates for Best Fitting Model With Age-Interaction Term: 2-Interior Knot Age Spline and Linear Age Interaction Term

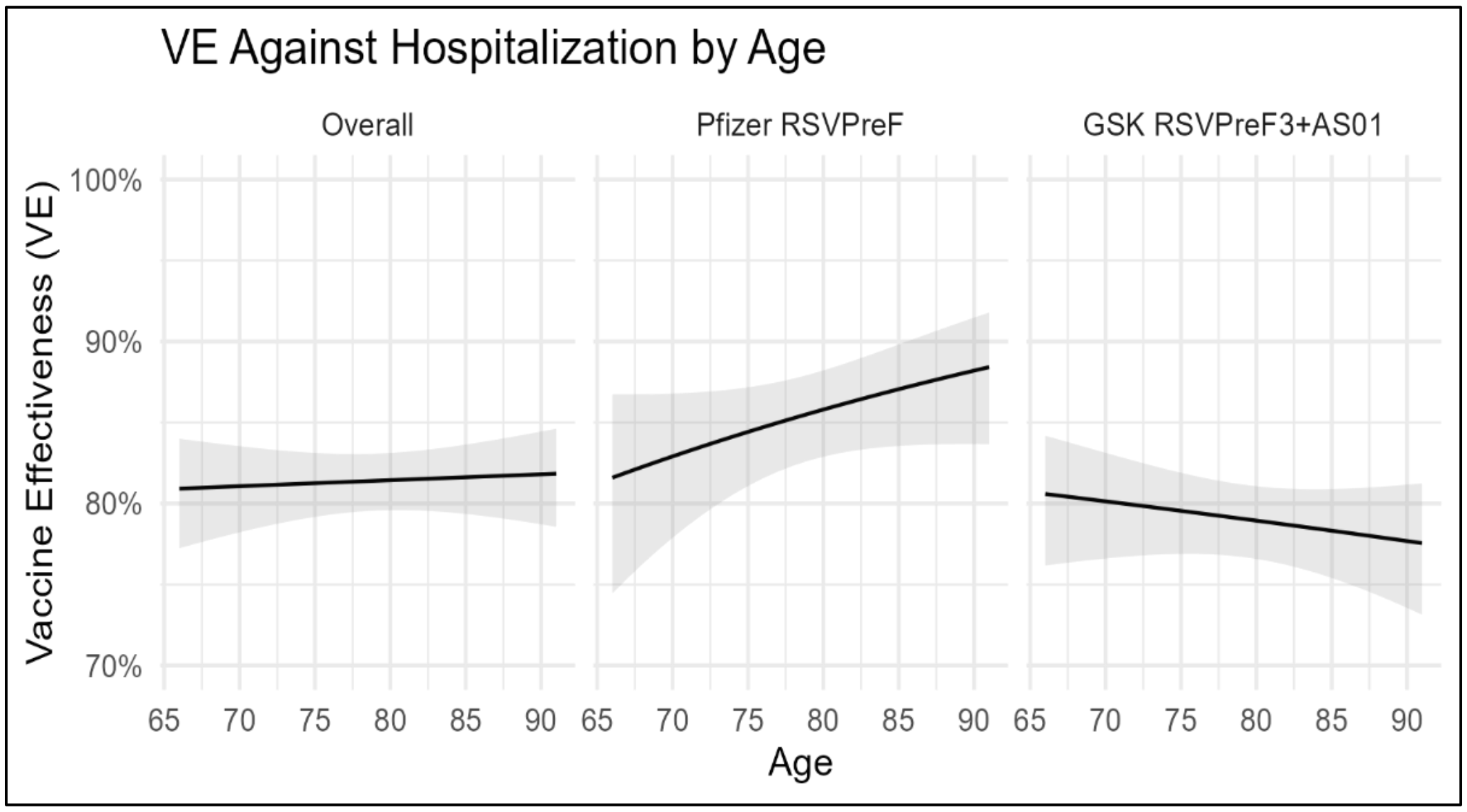

Note: The p-value when comparing the effect of age on VE between products was p = .068.

© 2026 Lu Y et al. *JAMA Network Open.*

S5. Immunocompromised Subgroup Results

eTable 7. VE Results by Immunocompromised Subcategories

Analysis	Unvaccinated Outcome Counts, Weighted				Vaccinated Outcome Counts, Weighted				VE
	Count	Person Weeks (100,000)	Rate per 100,000 Person Weeks	95% CI	Count	Person Weeks (100,000)	Rate per 100,000 Person Weeks	95% CI	RSV Vaccinated vs. Unvaccinated
Solid Malignancy	929	88.1	10.6	(9.9, 11.2)	58	19.1	3.0	(2.3, 3.8)	76.0% (68.3%, 81.8%)
Immunodeficiency (Treatment Required for Identification)	917	52.0	17.6	(16.5, 18.8)	62	12.4	5.0	(3.8, 6.2)	75.4% (68.0%, 81.1%)
Hematologic Malignancy	834	43.3	19.3	(18.0, 20.6)	74	10.3	7.2	(5.5, 8.8)	67.9% (58.7%, 75.1%)
Rheumatologic Inflammation	834	57.9	14.4	(13.4, 15.4)	59	13.4	4.4	(3.3, 5.5)	73.9% (65.8%, 80.1%)
Immunodeficiency (Diagnosis only Required for Identification)	646	26.8	24.1	(22.3, 26.0)	64	6.6	9.8	(7.4, 12.2)	64.6% (53.7%, 73.0%)
Transplant	263	8.4	31.4	(27.6, 35.2)	26	2.4	11.1	(6.9, 15.4)	69.1% (53.4%, 79.6%)
Solid Organ Transplant	159	7.1	22.5	(19.0, 26.0)	15	1.9	8.0	(3.9, 12.0)	68.2% (45.3%, 81.5%)
Stem Cell Transplant	167	2.7	62.0	(52.6, 71.4)	17	0.9	19.4	(10.1, 28.7)	70.4% (50.2%, 82.4%)

© 2026 Lu Y et al. *JAMA Network Open.*
