# Supplementary material for: Respiratory Syncytial Virus Vaccine Effectiveness Among US Older Adults, 2023-2024 Season
Source: JAMA Netw Open. 2026 Sep 18;9(9):e2634788. doi: 10.1001/jamanetworkopen.2026.34788 (PMC13589284; doi:10.1001/jamanetworkopen.2026.34788)
Supplement: Supplementary file 3 [file jamanetwopen-e2634788-s003.pdf]

Data Sharing Statement

Lu. Respiratory Syncytial Virus Vaccine Effectiveness Among US Older Adults, 2023-2024 Season. *JAMA Netw Open*. Published September 18, 2026.
doi:10.1001/jamanetworkopen.2026.34788

Data
Data available: No

Additional Information
Explanation for why data not available: All data produced in the present work are contained in the manuscript. Access to original datasets is not available to protect patient information.
